# Requirements for efficient ligand-gated co-transcriptional switching in designed variants of the *B*. *subtilis pbuE* adenine-responsive riboswitch in *E*. *coli*

**DOI:** 10.1371/journal.pone.0243155

**Published:** 2020-12-01

**Authors:** Lea K. Drogalis, Robert T. Batey

**Affiliations:** Department of Biochemistry, University of Colorado, Boulder, Colorado, United States of America; Arizona State University, UNITED STATES

## Abstract

Riboswitches, generally located in the 5’-leader of bacterial mRNAs, direct expression via a small molecule-dependent structural switch that informs the transcriptional or translational machinery. While the structure and function of riboswitch effector-binding (aptamer) domains have been intensely studied, only recently have the requirements for efficient linkage between small molecule binding and the structural switch in the cellular and co-transcriptional context begun to be actively explored. To address this aspect of riboswitch function, we have performed a structure-guided mutagenic analysis of the *B*. *subtilis pbuE* adenine-responsive riboswitch, one of the simplest riboswitches that employs a strand displacement switching mechanism to regulate transcription. Using a cell-based fluorescent protein reporter assay to assess ligand-dependent regulatory activity in *E*. *coli*, these studies revealed previously unrecognized features of the riboswitch. Within the aptamer domain, local and long-range conformational dynamics influenced by sequences within helices have a significant effect upon efficient regulatory switching. Sequence features of the expression platform including the pre-aptamer leader sequence, a toehold helix and an RNA polymerase pause site all serve to promote strong ligand-dependent regulation. By optimizing these features, we were able to improve the performance of the *B*. *subtilis pbuE* riboswitch in *E*. *coli* from 5.6-fold induction of reporter gene expression by the wild type riboswitch to over 120-fold in the top performing designed variant. Together, these data point to sequence and structural features distributed throughout the riboswitch required to strike a balance between rates of ligand binding, transcription and secondary structural switching via a strand exchange mechanism and yield new insights into the design of artificial riboswitches.

## Introduction

Riboswitches are RNA elements that adopt one of two mutually exclusive structures, dependent upon the occupancy status of a small-molecule binding domain, known as the aptamer domain, to direct expression of a message [1–3]. These structures generally inform transcription through formation of a Rho-independent (intrinsic) transcriptional terminator or translation by either occluding or exposing the ribosome binding site, although a diverse set of other mechanisms of expression regulation have been observed [4–6]. This straightforward mechanism of ligand-dependent control of gene expression without the assistance of accessory proteins has made these RNA-based input/output devices attractive for use in various synthetic biology applications [7,8]. In part, the means to raise RNA sequences that bind with high affinity and specificity to a broad spectrum of small molecules using *in vitro* selection approaches and the modularity of RNA secondary structure make the design and implementation of such input/output devices conceptually simple [9,10].

However, engineering modular riboswitches and other RNA devices has been problematic, such that only a few robust regulatory elements have achieved real-world application [8,11]. This suggests that a fundamental understanding of how these RNAs mechanistically translate ligand occupancy of the aptamer domain into an observable response remains incomplete. One major hurdle in the robust design of riboswitches is that they generally only function in the context of transcription [1,12–14], requiring careful consideration of kinetic processes such as rates of higher-order RNA folding, ligand binding and secondary structural switching processes such as strand exchange [15]. Furthermore, the influence of RNA sequence on processes such as transcriptional pausing and participation of factors such as NusA, NusG or Rho on riboswitches in the cellular context further confounds their design and implementation [16–18]. Some of these processes are directed by cryptic or context-dependent sequence elements that can be difficult to engineer into an RNA of interest.

**Fig 1:**
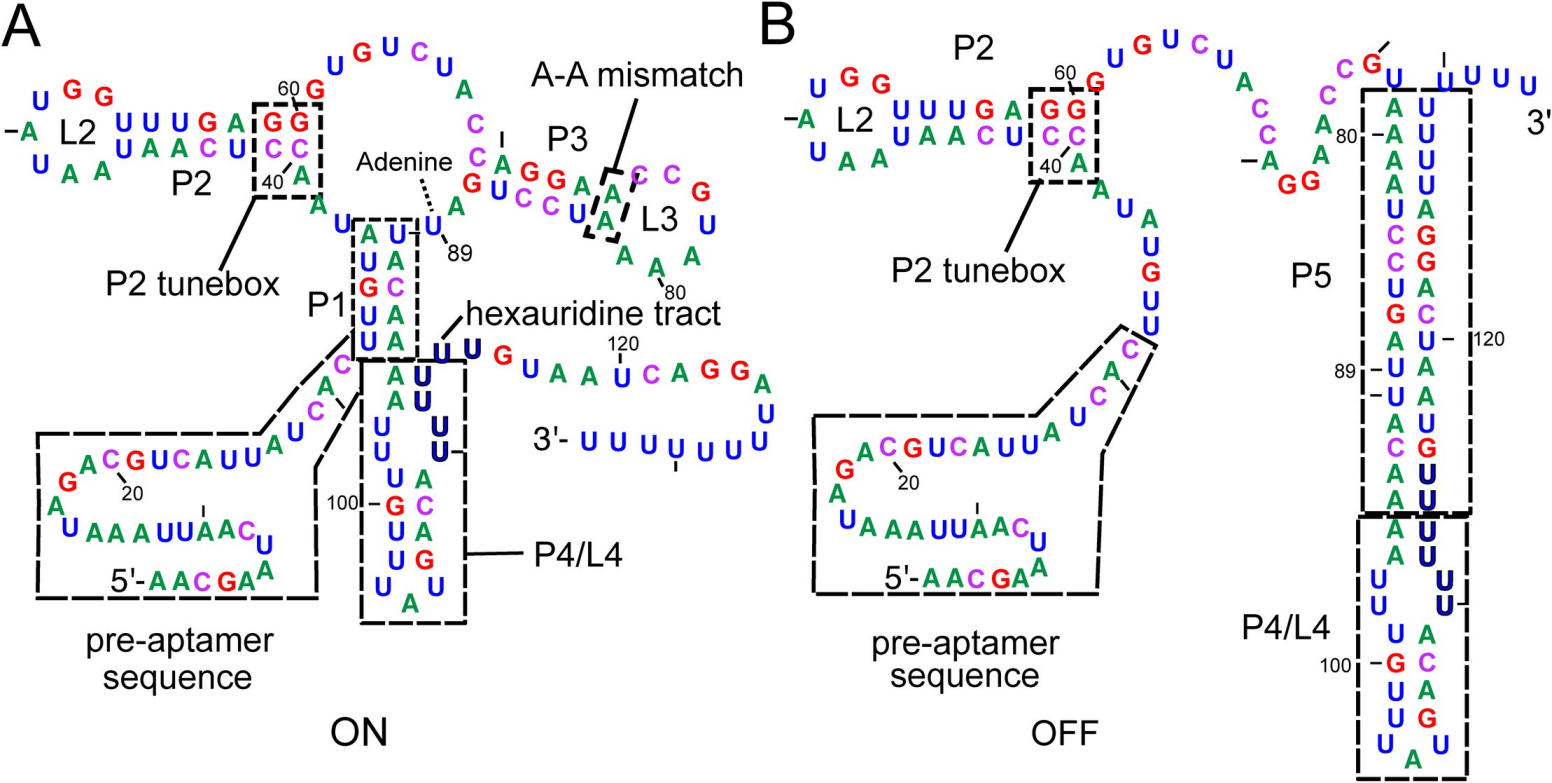
Secondary structures of alternative forms of the *pbuE* riboswitch. (A) Secondary structure of the *B*. *subtilis* adenine-responsive *pbuE* riboswitch starting at the transcriptional start site and ending at the poly-uridine tract in the ligand-bound state (ON). Relevant structural elements include the pre-aptamer leader sequence, P1 helix, P2 tunebox, P3 A-A mismatch and the P4/L4 stem-loop, highlighted in dashed boxes. The coloring scheme of this figure is used throughout this work to denote nucleotide identity; residue numbering reflects the experimentally determined start site of transcription [30]. (B) Secondary structure of the *pbuE* riboswitch in the unbound state (OFF) with the intrinsic terminator (P4/P5) formed at the expense of the P1 and P3 helices.

Natural riboswitches represent important model systems for understanding the sequence features that enable RNA input/output devices to function both *in vitro* and in the cell and inform how they can be engineered to be efficient ligand-dependent genetic regulators that function within a broad spectrum of industrially useful bacteria [19]. One of the simplest riboswitches containing a discrete ligand-binding domain and secondary structural switching domain (otherwise known as the expression platform) is the *Bacillus subtilis* adenine-responsive *pbuE* regulatory element (Fig 1) [20], making it ideal for detailed analysis of coupling of ligand binding to regulatory activity [21]. The aptamer domain shares the same general three-dimensional architecture as all purine riboswitches with the ligand binding site embedded within the three-way junction, adjacent to the 3’-side of the P1 helix [22,23]. Overall organization of the aptamer is achieved by a terminal loop-loop interaction (L2 and L3, Fig 1) whose formation is ligand-independent in some riboswitches such as the *B*. *subtilis xpt-pbuX* guanine-sensing riboswitch [24–26] and dependent on ligand binding in others such as the *pbuE* aptamer [27–29]. When ligand is bound within the three-way junction, the aptamer including the P1 helix is stabilized against formation of an alternative structure (P5, Fig 1) enabling full transcription of the mRNA and expression of the encoded purine efflux pump under high adenine conditions [20].

Single molecule analysis of the adenine-dependent folding of the *pbuE* riboswitch in the context of transcription has suggested a mechanism for how ligand binding to the aptamer domain prevents formation of the competing intrinsic terminator element [31]. In this model, during transcription the aptamer hierarchically and rapidly folds into a productive conformation by the time the polymerase reaches a hexauridine tract (nucleotides (nt) 100–105) found at the 3’-side of a stem-loop element (P4/L4) present in both the ON and OFF states of the riboswitch (Fig 1). Shortly thereafter, sequence on the 3’-side of P4 begins to strand exchange through the P1 helix. In the absence of ligand, this migration rapidly continues through J3/1 and P3 to form the functional terminator helix (P5) that prompts the RNA polymerase to disengage from RNA synthesis, and thereby gene expression is repressed. However, in the presence of ligand, strand exchange is impeded by ligand-dependent organization of the three-way junction and formation of base triples involving J2/3 and the two junction-proximal base pairs of P1. This set of triples acts as a ligand-dependent gate to prevent further strand exchange into the junction and P3, providing the RNA polymerase sufficient time to synthesize the message past the intrinsic terminator’s polyuridine tract and escape the riboswitch. Together with other biochemical and biophysical studies, there is compelling evidence that the *pbuE* riboswitch is a kinetically controlled switch such that it does not reach thermodynamic equilibrium with respect to adenine binding during the timeframe of transcription of the leader sequence [12,31,32]. Ultimately, the terminator form of the riboswitch represents the most thermodynamically stable state of RNA and at equilibrium the riboswitch adopts this structure regardless of ligand concentration [33].

Despite serving as a model system for understanding the physical basis for the coupling of binding and regulation, the *pbuE* riboswitch has not been extensively investigated in the cellular context. Because many riboswitches only function in the context of transcription and can be influenced by a number of cellular factors, understanding the sequence and structural requirements of the expression platform conferring ligand-dependent activity requires a cell-based reporter assay. Towards this end, a robust fluorescent protein reporter assay was previously developed for the *pbuE* riboswitch using the adenine analog 2-aminopurine (2AP) as the ligand for use in the heterologous organism *E*. *coli* [34]. This study revealed several facets of the expression platform that influence ligand-dependent regulatory activity. The most important finding was that the riboswitch is highly tolerant to significant variation in the length of the P1 helix. This contrasts results of biophysical studies which proposed that P1 helix length and associated thermodynamic stability are critically important for proper switching [35]. Instead, this result supports a low-energy and kinetically rapid strand displacement mechanism that governs formation of the riboswitch’s alternative secondary structures [34]. Second, these data suggested that the ligand-independent hairpin P4/L4 plays an important role in regulatory activity. This indicates that sequences and secondary structures within the expression platform influence the regulatory response.

In the current study, we seek to further define regions of the *pbuE* riboswitch that influence the ligand-dependent regulatory activity using a structure-guided mutagenic approach. To assess whether the aptamer domain has features beyond the conserved ligand binding site that are required for efficient secondary structural switching, a set of *B*. *subtilis* purine-responsive aptamers was spliced into the *pbuE* expression platform to create chimeras homologous to the native *pbuE* riboswitch. Despite very high sequence and structural similarities, these designed chimeras fail to function in *E*. *coli*. Regulatory activity is restored by introducing point mutations in two regions that influence the conformational ensemble of the unliganded state, suggesting new roles for sequences in the aptamer domain. To further clarify structural effects on strand exchange, we examined the sequence of the P1 helix, the P4/L4 hairpin, and a putative programmed pause. These mutants revealed altered regulatory properties that indicate pausing and formation of a “toehold” by P4 is essential for regulatory activity, while the sequence of the P1 helix is likely tuned to influence the rate of strand invasion immediately prior to the key regulatory roadblock. Together, these data reveal new aspects of how the riboswitch’s activity is modulated by key sequence and structural elements and suggest new design strategies for the engineering of novel riboswitches.

## Materials and methods

### Construction of reporter plasmids

For each riboswitch, a set of overlapping DNA oligonucleotides was synthesized to construct the gene using recursive PCR [36]. Using standard molecular cloning techniques [37], each riboswitch variant was cloned upstream of the *gfpuv* gene to regulate its expression in a ligand-dependent manner. The parental vector containing *gfpuv* is derived from a low-copy pBR327 plasmid [38] and contains the strong *rrnB* terminator upstream of a synthetic insulated promoter [39] of moderate strength to limit transcription readthrough from upstream genes. All plasmids were sequenced verified. S1 Table provides the sequences of each riboswitch used in this study from the first transcribed nucleotide to the polyuridine tract.

### Cell-based fluorescence assays

*E*. *coli* K12 strain BW25113 (Keio knockout collection parental strain) [40] was transformed with reporter plasmid using standard protocols [37]. Transformants were plated on 2xYT (Research Products International) growth medium agar plates containing 100 μg/mL carbenicillin for resistance marker selection. A single colony was picked and used to inoculate an overnight 2 mL outgrowth culture of 2xYT containing 100 μg/mL of ampicillin and grown at 37°C. A 20 μL volume of overnight culture was used to inoculate three individual 2 mL cultures of a chemically defined growth (CSB) medium [38] containing 100 μg/mL of ampicillin. Outgrowth of *E*. *coli* in either 2xYT or CSB medium did not affect experimental results, and therefore 2xYT was used for outgrowth prior to each experiment and that the 2 mL experimental culture contained a 1:100 dilution of exhausted 2xYT medium from the outgrowth. Three additional 2 mL CSB plus ampicillin cultures containing 1 mM 2-aminopurine (2AP) were also inoculated with saturated overnight cultures in a 1:100 dilution. These secondary cultures were grown to an OD_600_ between 0.3–0.5 at 37°C. The growth rates of cells in the presence and absence of 2AP in CSB medium were similar for E. coli BW25113 transformed with empty vector (pBR322) or reporter vectors (S1 Fig). Expression was determined by measuring the O.D. (600 nm) (cell density) and fluorescence intensity of 175 μL of each culture using an Infinite M200 PRO plate reader (Tecan). Cells were excited at 395 nm and read at 510 nm for all fluorescence measurements with maximum fluorescence being set with a well containing 175 μL of 3 μM fluorescein standard. Normalized fluorescence per cell density was calculated by dividing fluorescence measurements by the corresponding cell density values. Fold induction was calculated by subtracting background fluorescence (normalized fluorescence of cells containing only parental vector) from normalized fluorescence values and fluorescence values of cultures containing ligand divided by those without ligand. Data for each biological replicate were collected on different days to account for day-to-day variation; all data are based upon at least three technical repeats of three independent biological replicates (S2 Table). Standard errors (S2 Table) were calculated using Excel (Microsoft). t probability values (*p*) were calculated using an unpaired t-test assuming normally distributed populations with unequal variances implemented in KaleidaGraph (v. 4.1, Synergy Software).

### Co-transcriptional RNA folding simulations

The co-transcriptional folding landscapes of select mutant riboswitches were modeled using Kinefold (http://kinefold.curie.fr; [41]) Riboswitch RNA sequences starting at the transcription initiation site and ending after the polyuridine tract of the Rho-independent transcriptional terminator were used in the simulation with the transcriptional speed set as a new nucleotide added every 20 milliseconds (50 nt sec^-1^) with pseudoknots disallowed. Helical tracing graphs were obtained from these simulations as well as folding movies showing the lowest free energy structure with the addition of each base. The resultant folding trajectories allow for visual determination of persisting secondary structures that are not reported in the helical folding graphs.

## Results and discussion

### Modified purine aptamer domains direct the *pbuE* expression platform

To facilitate construction of the various riboswitch variants in this study, the pre-aptamer sequence (Fig 1) of wild type *pbuE* riboswitch was modified by deletion of the first eleven nucleotides (Δ11) and addition of an *Aat*II restriction site the resultant pre-aptamer sequence and a *Spe*I site immediately after the polyuridine tract of the intrinsic terminator (variant Δ11/RS, S1 Table). These changes result in a small, but statistically significant (*p* < 0.05) improvement in the 2AP-dependent fold induction of the riboswitch and was also comparable to an almost complete deletion of the pre-aptamer sequence (Δ27) (S2 Fig). These observations are consistent with previous observations that the length of the pre-aptamer sequence minimally impacts the magnitude of 2AP-mediated induction of expression of the fluorescent reporter gene. The redesigned (Δ11,RS)*pbuE* riboswitch is referred to as the pbuE* variant in this work.

It has been hypothesized that some expression platforms are modular and capable of hosting different aptamers to create chimeric riboswitches with new activities. This has been demonstrated experimentally for both OFF and ON switches using a single-turnover *in vitro* transcription assay and cell-based reporters [38,42,43]. A significant limitation in the use of modular domains to engineer novel riboswitches is that the secondary structural switch must be constrained to the expression platform. In the case of the *pbuE* riboswitch, the secondary structural switch involves sequence elements from both the aptamer domain and the expression platform (Fig 1). This makes the *pbuE* expression platform incompatible with other aptamer modules since sequence from the aptamer domain is required to form the terminator element. One approach to circumvent this issue was engineering the expression platform to not strand invade into the aptamer domain to form the intrinsic terminator to facilitate the “mix-and-match” strategy [38]. Using this approach, a chimeric riboswitch using *B*. *subtilis xpt-pbuX* guanine/hypoxanthine-responsive riboswitch aptamer domain [22] with the *B*. *subtilis pbuE* adenine-responsive riboswitch expression platform achieved ~14-fold induction of reporter expression in *E*. *coli* [38]. While this strategy was successful across a number of aptamer domain/expression platform pairings *in vitro*, these engineered riboswitches performed poorly in *E*. *coli*. An alternative design approach would be to alter the sequence of the expression platform module to enable strand invasion through the aptamer domain to form the terminator element in the absence of ligand.

**Fig 2:**
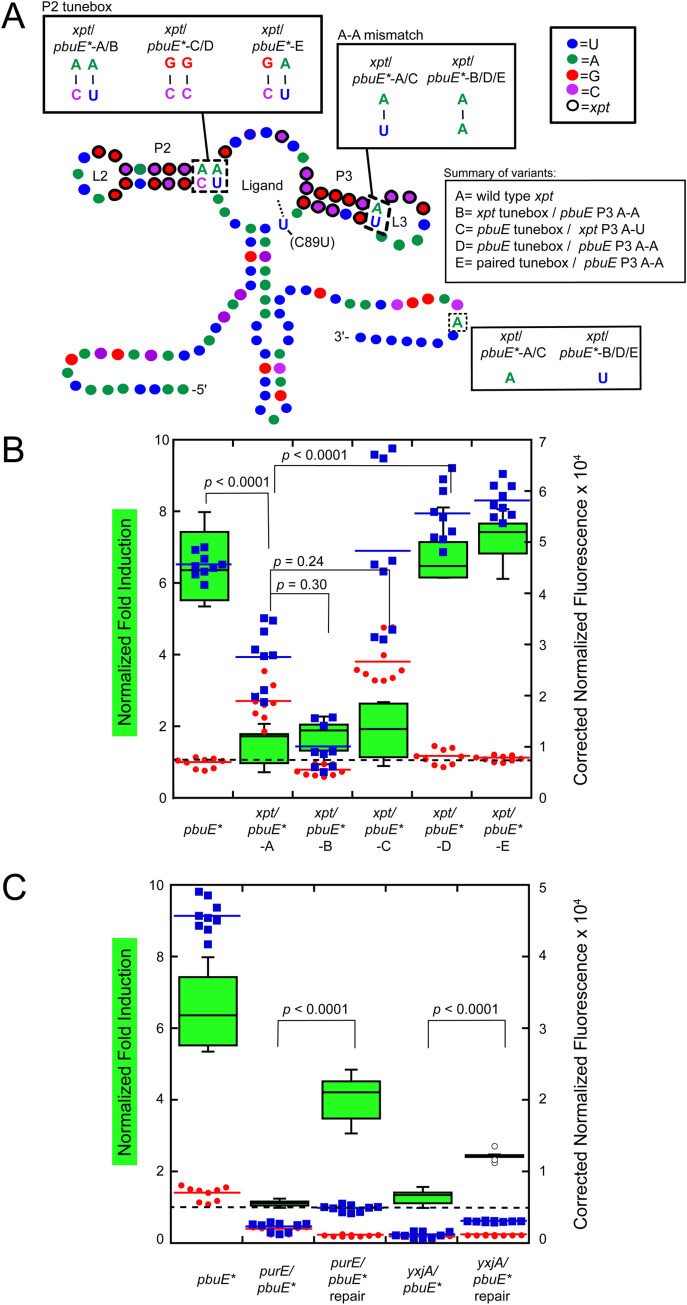
Multiple mutations are required for switching of the *xpt*(C89U)/pbuE* chimera. (A) Secondary structure of the *xpt*(C89U) aptamer appended to the expression platform of the pbuE* riboswitch. The pre-aptamer sequence has been omitted for clarity. Relevant structural elements include the P2 tunebox and the P3 distal A-A mismatch. The nucleotide coloring scheme is the same as in Fig 1; residues circled in black are part of the *xpt*(C89U) aptamer. (B) Box-and-whisker plot of the fold induction for each riboswitch variant tested in which the central bar represents the median fold induction value (left y-axis), the green box represents the interquartile range and the bars represent the spread; n ≥ 9 measurements for each variant. Background corrected normalized fluorescence data for each *xpt*(C89U)/pbuE* chimera variant in the absence (red) and presence (blue) of 2AP (right y-axis). Each data point represents one of three technical replicates of three biological replicate; the colored bar represents the median value of the biological replicates. *p*-values relate to the comparison of the fold induction values of two variants. Dotted line indicates no induction (fold induction equals one). (C) Fold induction and background corrected normalized fluorescence data for *purE*(C89U)/pbuE* and *yxjA*(C89U)/pbuE* chimera variants in the absence (red) and presence (blue) of 2AP. “Repair” variants contain mutations to the tunebox and/or P3 helix that restored activity to the *xpt*(C89U)/*pbuE* chimera.

As a first step to address this approach, we reinvestigated a chimeric riboswitch in which the *B*. *subtilis xpt-pbuX* guanine/hypoxanthine-responsive riboswitch aptamer domain [22] was coupled the *B*. *subtilis pbuE* adenine-responsive riboswitch expression platform (Fig 2A). For these studies, the C89U mutation within the *xpt* aptamer was used that switches the aptamer’s specificity to adenine/2-aminopurine (note that in the context of the *xpt* riboswitch this nucleotide is at position 74 and is referred to as the C74U mutation, but in this work this position is numbered 89 to be consistent with *pbuE* numbering, Figs 1 and 2A). This mutant has been extensively used to study the *xpt* aptamer and does not alter the properties of this RNA [44–46]. While only P2 and P3 are highlighted in Fig 2A, the junction-proximal two base pairs of P1 as well as all nucleotides in J1/2 and J3/1 are identical between the *xpt*(C89U) and *pbuE* aptamers along with seven out of eight nucleotides in J2/3. The only nucleotide difference in J2/3 is G56 of *pbuE*, the equivalent position being uridine in *xpt*. Thus, the primary differences in these aptamer domains lie in P2/L2 and P3/L3. To make the *pbuE* expression platform compatible with the *xpt* aptamer domain, compensatory changes were made within the expression platform to enable formation of a stable transcriptional terminator hairpin with full canonical base pairing of the same length as that of the wild type *pbuE* riboswitch (Fig 2A).

As the secondary and tertiary structure of all purine riboswitches is highly conserved, we expected that this chimera (xpt/pbuE*-A) would robustly turn on reporter protein expression in the presence of 2AP. However, compared to the pbuE* riboswitch, the chimera exhibited no activation of gene expression (*p* < 0.0001, Fig 2B). Notably, the chimera has elevated reporter expression in the absence of 2AP as compared to pbuE* (*p* < 0.0001), suggesting that the intrinsic terminator has difficulty forming efficiently (red circles, Fig 2B). To understand why this chimeric RNA fails to regulate gene expression, a mutational analysis of the aptamer domain was performed in which elements of the *xpt*(C89U) P2 and P3 were systematically substituted back for the *pbuE* sequences. This survey revealed that that two specific point substitutions made in the *xpt*(C89U) aptamer resulted in robust 2AP-dependent gene regulation.

The first region affecting regulatory activity of the *xpt*(C89U)/pbuE* chimera is the base pair in P3 proximal to L3. To achieve the OFF state, the *pbuE* terminator must invade into L3, disrupting the stable L2-L3 interaction. Single molecule and fluorescence lifetime studies demonstrated that while the *xpt* L2-L3 interaction is stable in the absence of ligand [25,26,29], the equivalent interaction in *pbuE* is only transiently stable in the absence of ligand binding [27,28]. Further, introduction of an A-A mismatch as seen in *pbuE* at the base of the *xpt* L3 destabilizes the L2-L3 interaction in a single-molecule study [24]. Introducing a point substitution into the *xpt*(C89U)/pbuE* hybrid to yield an A-A mismatch (xpt/pbuE*-B) was insufficient to restore regulation (Fig 2B). However, this substitution restored efficient terminator formation, as evidenced by low reporter expression in the absence of 2AP, indicating that destabilizing the L2-L3 interaction is important to enable full strand exchange through L3.

The second region of the aptamer that may affect regulatory activity is the P2 tunebox, which comprises the junction-proximal two base pairs in P2 helix along with the last nucleotide of J1/2 (Fig 1A) [46]. Natural variation in this region of the purine riboswitch aptamer has a significant effect on its kinetic and thermodynamic ligand binding properties. It was hypothesized based upon chemical probing data that this region affects the organization of the three-way junction in the absence of effector ligand [46]. However, introducing a tunebox sequence identical to that of *pbuE* into the *xpt*(C89U)/pbuE* chimera (xpt/pbuE*-C) alone was also insufficient to restore switching ability (Fig 2B).

Instead, simultaneous introduction of the *pbuE* P2 tunebox and the L2-L3 destabilization element into the hybrid (xpt/pbuE*-D) was required to restore switching activity to that of wild type level (Fig 2B). Furthermore, simply replacing the non-canonical C-A base pair in the *xpt* aptamer domain P2 to a canonical C-G pair (xpt/pbuE*-E*)* also restores switching performance and improves leaky expression in the absence of 2AP when introduced in concert with the distal A-A mismatch. These data strongly suggest the conformational dynamics of the aptamer and associated crosstalk between the L2-L3 interaction and the three-way junction in the unliganded state are critical for establishing a strong ligand-dependent regulatory response.

To determine whether these two features of the *xpt* aptamer also influence the ability of other guanine-binding aptamers to regulate the *pbuE* structural switch, we investigated several other chimeras. Using the same strategy as with *xpt* to create a 2AP-responsive chimera, the *B*. *subtilis purE* (S3 Fig) and *yxjA* (S4 Fig) guanine riboswitch aptamer domains [47] were placed into the pbuE* expression platform. The wild type *purE* riboswitch aptamer domain lacks both the P3 A-A mismatch and a fully base paired P2 tune box, leading to the design of a “repaired” *purE* with the same mutations as the optimal xpt/pbuE*-E construct (*xpt* A-A P3 plus paired tune box). Consistent with *xpt*(C89U)/pbuE*, the *purE*(C89U)/pbuE* hybrid did not show any switching activity (Fig 2C) and gene expression in the presence or absence of 2AP was low. The repaired *purE*(C89U)/pbuE* hybrid showed improved termination in the absence of 2AP and increased aptamer stability in the presence of 2AP with a 4-fold induction of reporter expression (Fig 2C). Switching performance was not as effectively repaired in the case of *purE*(C89U)/pbuE* compared to *xpt*(C89U)/pbuE*, but a significant restoration of regulatory activity was still observed.

Unlike the *purE* guanine-sensing aptamer, the wild type *yxjA* aptamer contains a P3 A-A mismatch at the base of L3 as well as a P2 tune box with two canonical base pairs like the *pbuE* aptamer. Thus, this aptamer was anticipated to work with the *pbuE* expression platform and yield effective switching without altering its sequence. Surprisingly, the yxjA(C89U)/pbuE* chimera displayed little 2AP-dependent induction of expression (Fig 2C). A variant of *yxjA* containing the tune box sequence identical to the *pbuE* aptamer was also examined; this repaired *yxjA*(C89U)/pbuE* chimera showed only modest switching activity, though expression increased in both the presence and absence of 2AP relative to the unrepaired chimera. Together, these data suggest that two elements within the P2 and P3 helices of the purine aptamer domain that often harbor base mismatches, while not directly involved in ligand binding are nonetheless critical for establishing a regulatory response. However, it is likely there remain other still unidentified features in the aptamer domain that influence regulatory activity since the performance of these two chimeras was worse than that of the *xpt* chimera both with respect to overall fold induction and levels of reporter expression when induced with 2AP (Fig 2B).

### Base pair identity within the P1 helix does not correlate with regulatory activity

**Fig 3:**
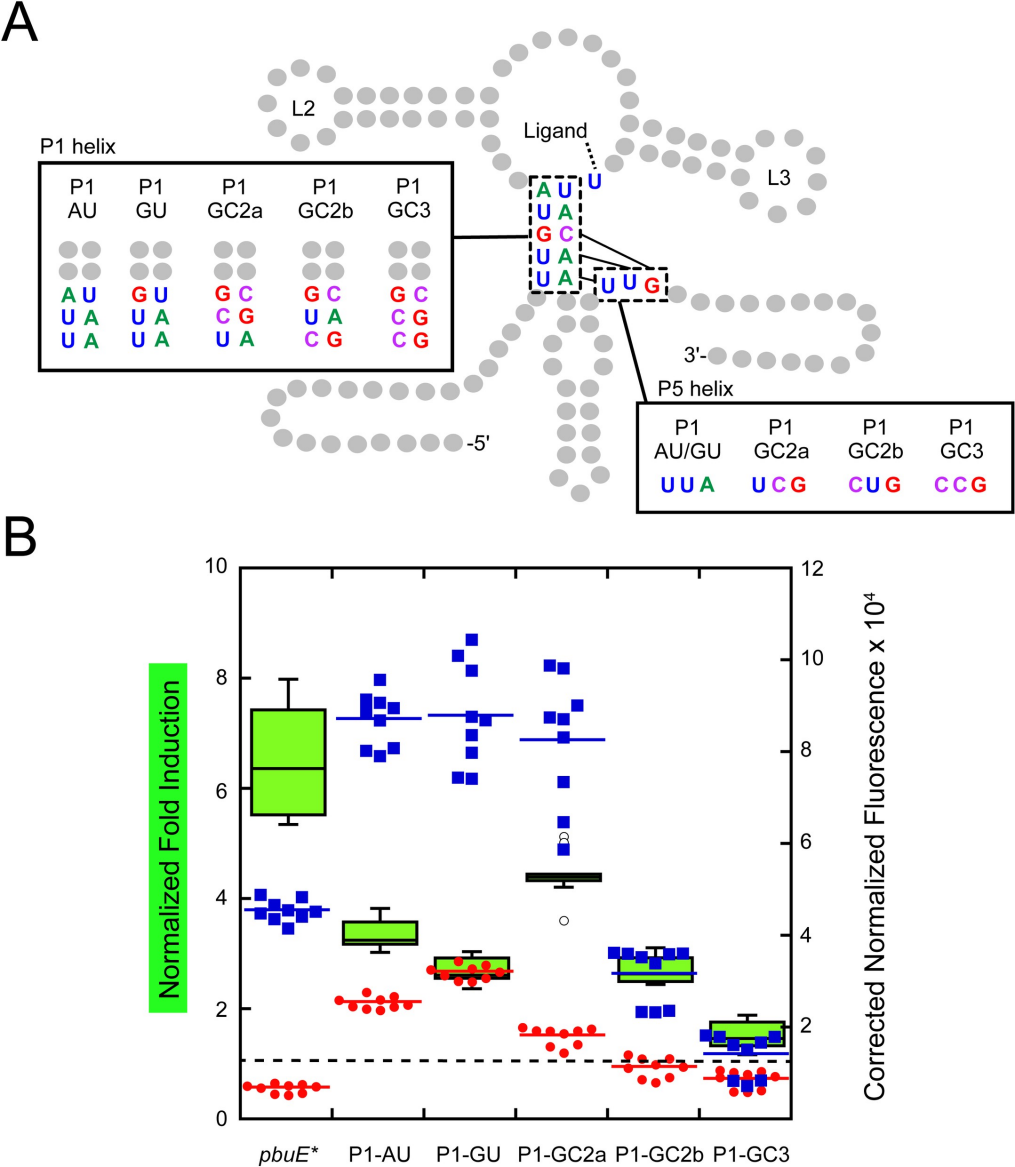
Altering P1 sequence impacts regulatory activity. (A) Secondary structure of the *pbuE* riboswitch highlighting each mutant of the P1 helix. Relevant structural elements include the P1 helix and the P1 helix pairing region of the expression platform. (B) Fold induction (left x-axis) and normalized fluorescence data for each P1 helix mutant in the absence (red) and presence (blue) of 2AP (right x-axis) using same display format as in Fig 2. The dotted line indicates a fold induction of one. Open circles represent fold induction measurements considered to be outliers (calculated as a value greater than either the lower or upper quartile value plus 1.5×interquartile distance) but still included in the dataset.

It is also likely that the sequence of the P1 helix—a participant in the regulatory switch—has a significant role in promoting the ligand-dependent structural switch. In a prior study, it was proposed that the sole G-C base pair in the wild type *pbuE* P1 helix represents an important road block to rapid invasion through P1, giving the aptamer sufficient time to bind effector [48]. To directly test this hypothesis, mutations were made to P1 that either replace the G-C pair with an A-U pair or add G-C pairs to the junction-distal region of the P1 helix (Fig 3A). The two junction proximal base pairs were not altered since they form ligand-dependent base triples with nucleotides in J2/3. Mutations designed to weaken the P1 helix were anticipated to decrease expression in the absence of ligand due to the increased ease of strand invasion by the expression platform through P1, which would improve the observed fold-induction. Conversely, adding G-C pairs to P1 was expected to decrease expression in the absence of ligand as strand invasion through P1 is impaired thereby diminishing the magnitude of 2AP-induced reporter expression. In contrast to these expectations, the P1-AU and P1-GU variants have clearly higher levels of reporter expression in both the absence and presence of 2AP, suggesting that strand invasion is impaired relative to wild type (red circles, Fig 3B). Additionally, while all of the P1 variants displayed reduced magnitude in 2AP-dependent induction, there is no clear correlation between the G-C content of P1 and fold induction. Nonetheless, it appears that base pair identities in P1 impact efficient strand exchange.

### Altering the pre-aptamer leader sequence unmasks a strong influence of P1 sequence on regulatory activity

One possibility for the lack of a clear trend in fold induction within the above set of P1 variants is that sequence changes in P1 result in alternative structure(s) that interfere with formation of functional structures. In some natural riboswitches, this sequence can have important roles in the RNA’s function and thus cannot be entirely dismissed. In the case of the *Vibrio vulnificus add* riboswitch, the 5’ region of the mRNA forms participates in an alternative secondary structure the promotes temperature sensing [49], while in the *Clostridium beijerinckii* ZMP riboswitch an intermediate hairpin forms that facilitates folding of the aptamer [50]. To explore the potential of alternative aptamer folding in designed variants, a subset of sequences was analyzed by Kinefold, a program that simulates co-transcriptional folding pathways [41]. Analysis of the wild type *pbuE* riboswitch used in this study indicates a pathway in which P2 and P3 fold, followed by a brief formation of P1 that is then disrupted by folding of the terminator, which is consistent with experimentally derived models of the *pbuE* folding pathway [31,48]. However, in both the P1-GC2b and P1-GC3 variants, the helical formation traces revealed the appearance of alternate pairing schemes involving the 5’-side of P1 and the pre-aptamer leader sequence, indicating alternative folds disruptive to the P1 helix and aptamer formation.

**Fig 4:**
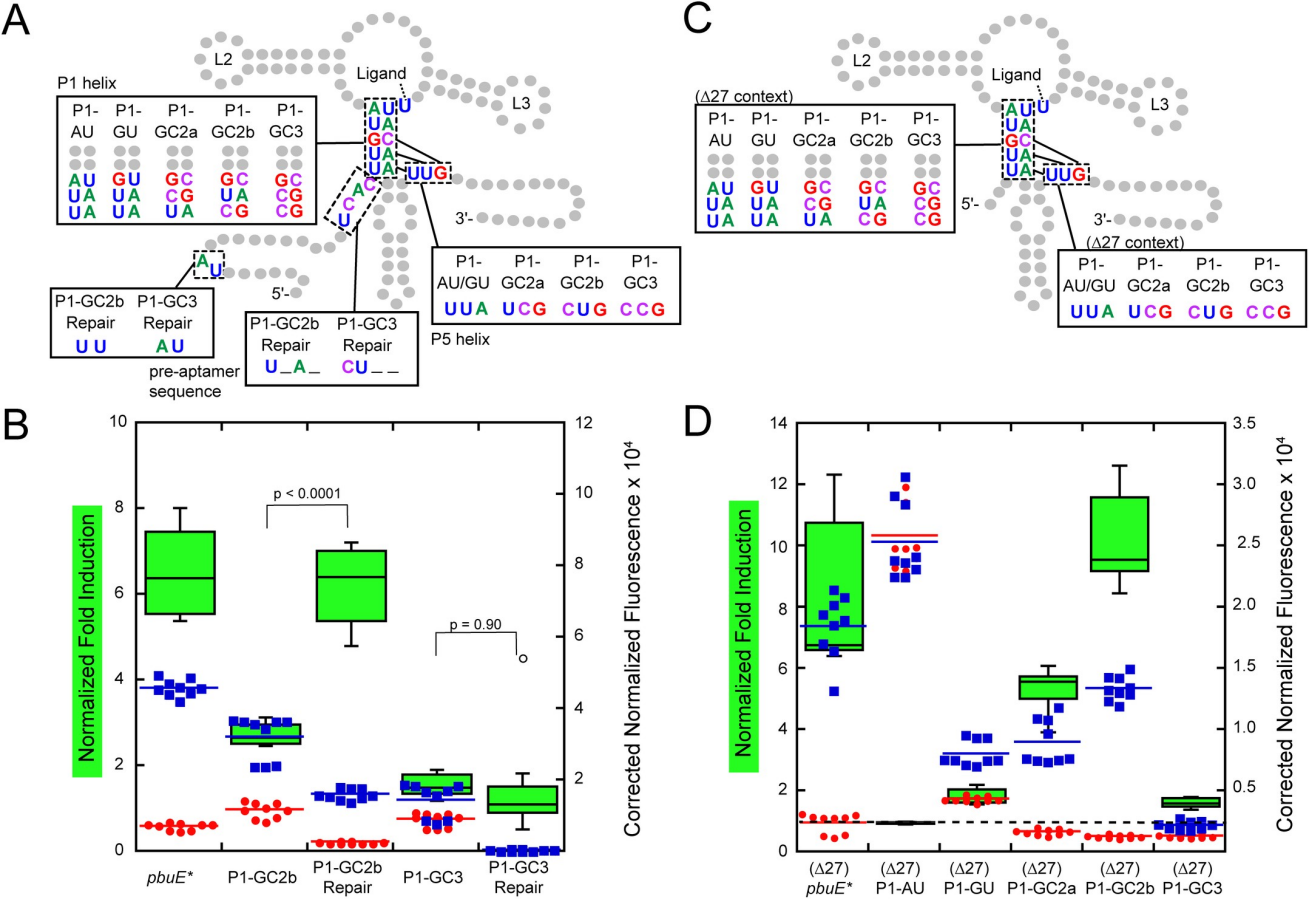
Removal of the pre-aptamer sequence helps clarify trends of P1 variants. (A) Secondary structure of the *pbuE* riboswitch including mutations in the leader sequence designed to ablate unintended pairing interactions. Mutations in the P1 helix are highlighted along with the compensatory changes required in the expression platform. (B) Fold induction and normalized fluorescence data for the repair variants (“Repair”) in the absence (red) and presence (blue) of 2AP along with wild type and unrepaired variants. Dotted line indicates a fold induction of one. (C) Secondary structure of the *pbuE* riboswitch in which the first 27 nucleotides of the pre-aptamer sequence are deleted (referred to as Δ27). Mutations in the P1 helix are highlighted along with the compensatory changes required in the expression platform. (D) Fold induction and normalized fluorescence data for each P1 helix mutant without the leader sequence in the absence (red) and presence (blue) of 2AP.

With this hypothesis in mind, these two variants were redesigned to minimize alternative pairing. Using Kinefold simulations, the pre-aptamer sequence was altered to prevent alternative pairing with P1 helical elements (P1-GCb,rep and P1-GC3,rep). These “repair” variants (Fig 4A) show different regulatory properties. Repairing the mismatched folding in P1-GC2b was sufficient to both improve the fold induction to the level of wild type *pbuE* and substantially improve the ability of the terminator to form in the absence of 2AP. Notably, only a few point substitutions of the pre-aptamer sequence that destabilize the predicted alternative helix are sufficient to restore activity (Fig 4B). Conversely, repair of P1-GC3 did not greatly improve switching, instead promoting strong terminator formation in both the absence and presence of 2AP. These data indicate that while the pre-aptamer leader sequence in the context of the wild type riboswitch is neutral, it can confound mutagenic analysis of riboswitch function or variant design via the formation of unanticipated alternative structures.

To resolve the issue of mutation-induced misfolding by the pre-aptamer sequence, a new set of P1 mutants was designed in which the pre-aptamer sequence was almost completely removed through a deletion of nucleotides 1–27 (referred to as Δ27) of the wild type full length sequence (Fig 4C). This deletion in the context of the wild type riboswitch did not substantially alter its regulatory properties, although a small increase in the 2AP-dependent reporter gene expression was noted (S3 Fig). In the absence of the pre-aptamer sequence, a clear trend in the function of the P1 helix variations was observed (Fig 4D). Variations that weaken the P1 helix, (Δ27)P1-AU and (Δ27)P1-GU, showed no or very little ability to activate gene expression in the presence of 2AP. Most notably, (Δ27)P1-AU displayed a high level of reporter expression in both the presence and absence of 2AP, suggesting that the terminator does not efficiently invade through the aptamer even in the absence of ligand binding. Variant (Δ27)P1-GU is still able to terminate, but not as well as variants containing G-C pairs in the P1 helix. Together, these two variants suggest that the weakened P1 helix (and weakened terminator helix in the complementary region) does not promote full strand exchange on the timescale that enables the regulatory switch. Variants that stabilized the P1 helix by adding another G-C base pair ((Δ27)P1-GC2a and (Δ27)P1-GC2b) display expression levels and fold-induction comparable to wild type, indicating that the riboswitch accommodates a more G-C rich P1 helix. However, further addition of a third G-C pair to the P1 helix ((Δ27)P1-GC3) yields an RNA that is constitutively terminated, indicating that a P1 helix rich in G-C base pairs likely supports rapid strand exchange to form the terminator helix at the expense of the aptamer having sufficient time to bind 2AP. Together, these data strongly argue that the base pair composition of helices through which strand exchange occurs has a critical effect on the regulatory activity of a riboswitch.

### The P4/L4 stem-loop is critical for secondary structural switching

**Fig 5:**
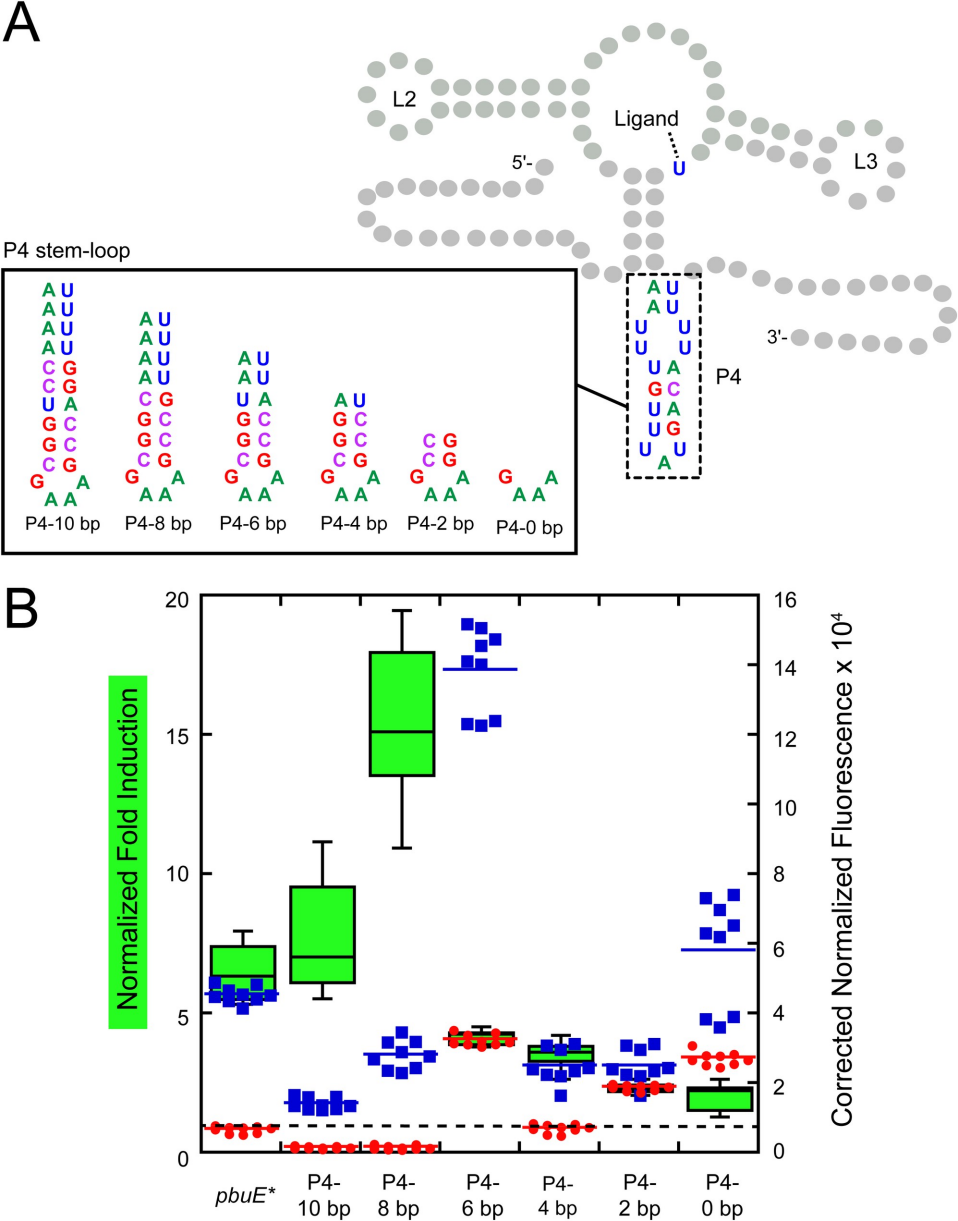
P4 length impacts the efficiency of regulatory activity. (A) Secondary structure of the *pbuE* riboswitch highlighting each P4/L4 hairpin variant. The pre-aptamer sequence is that of pbuE*. (B) Fold induction and background corrected normalized fluorescence data for each stem-loop variant in the absence (red) and presence (blue) of 2AP. Dotted line indicates a fold induction of one.

In a strand exchange model of ligand-dependent switching, an expression platform alternative secondary structure must rapidly begin the process of strand exchange with some component of the aptamer domain. It has been observed that riboswitch expression platforms often contain a hairpin element like P4/L4 in the *pbuE* riboswitch that can be formed in either conformational state that may help initiate (Fig 1A) strand exchange [34,51]. Like related toehold-mediated strand displacement processes [52,53], the kinetics of invasion is likely related to the length and/or base pair composition of this element. To examine the importance of the P4 stem-loop length on regulatory activity, the wild type stem-loop was replaced with a set of designed helices of varying lengths capped with a stable GAAA tetraloop to promote hairpin folding (Fig 5A). This series spans from 0 to 10 canonical base pairs in length (note that the wild type P4 helix is 8 base pairs including two U-U mismatches) with the hairpin sequence designed to maximize the size of the uridine track at its 3’-side.

This series of RNAs displays a trend towards increased fold induction in the presence of 2AP as the length of the P4 helix increases with one clear exception (Fig 5B). As the length of the nucleator helix increases from 0 to 8 base pairs, the fold induction increases from 2- to over ~15-fold, indicating that increasing helix length has a positive effect on the regulatory activity. Further increasing the length of P4 from 8 to 10 base pairs reduces the fold induction, but to no worse than wild type. In both the P4-8 bp and P4-10 bp variants, expression levels in the absence of 2AP are extremely low (red circles, Fig 5B) suggesting that they promote efficient formation of the terminator element. Surprisingly, regulatory activity is not completely abolished in the NH-0 variant, indicating that P4 is not absolutely essential for activating gene expression, but without this element strand invasion is impaired. A notable exception in this trend is P4-6 bp which exhibits higher expression in the absence and presence of 2AP, indicating that this variant also has an impaired ability to form the terminator element. Thus, while the length of the P4 helix promotes strong regulatory activity, other elements in this region of the expression platform are important for this process.

A second feature of P4 that may impact regulatory activity is a potential hexauridine “programmed pause” at the 3’-side of the helix. Many riboswitches contain stretches of uridines in their expression platforms that represent RNA polymerase pause sites that give more time for events such as RNA folding and ligand binding to occur [13,17,54,55]. In the *pbuE* riboswitch, the P4 hexauridine tract has been observed to be a pause site *in vitro* using *E*. *coli* and *B*. *subtilis* RNAP [30], but was not identified as a pause site in two genome-wide surveys of transcriptional pausing in *B*. *subtilis* [56,57]. In the design of the P4 length variant series, only P4-8 bp and P4-10 bp retain the hexauridine tract; the other variants reduced or eliminate this tract.

**Fig 6:**
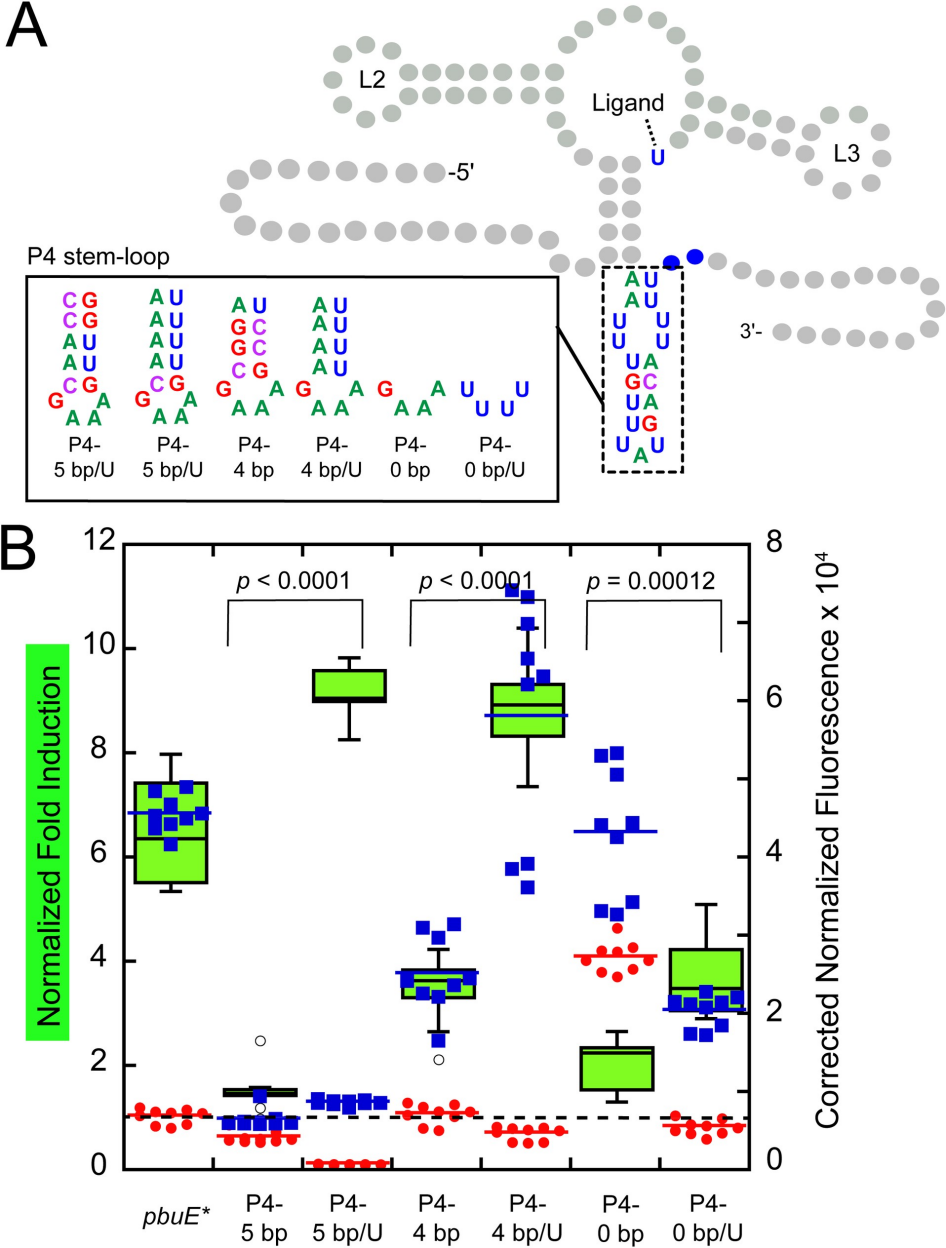
The hexauridine tract is important for efficient regulatory activity. (A) Secondary structure of the *pbuE* riboswitch highlighting each P4-5 bp, P4-4 bp, and P4-0 bp variants; the aptamer domain remains unchanged. (B) Normalized fold induction and background corrected normalized fluorescence data for each variant in the absence (red) and presence (blue) of 2AP. Dotted line indicates a fold induction of one.

To determine the importance of this pause in *E*. *coli*, a set of variants were designed that preserve the six uridines on the 3’-side, including a variant with a five base pair helix (P4-5 bp and P4-5 bp/U) and variants of NH-4 and NH-0 from the original nucleator length series (P4-4 bp/U and P4-0 bp/U) (Fig 6A). In all three variants, the presence of a hexauridine tract significantly improved 2AP-depedent activation of gene expression (Fig 6B). Additionally, in the absence of 2AP, all variants containing a hexauridine tract show reduced reporter expression, indicative of more efficient strand invasion to form the terminator. Together, these data argue that the hexauridine tract is important for modulating the temporal window of transcription to enable key events more time to occur.

### An elemental pause and toehold are the critical features of stem-loop P4/L4

**Fig 7:**
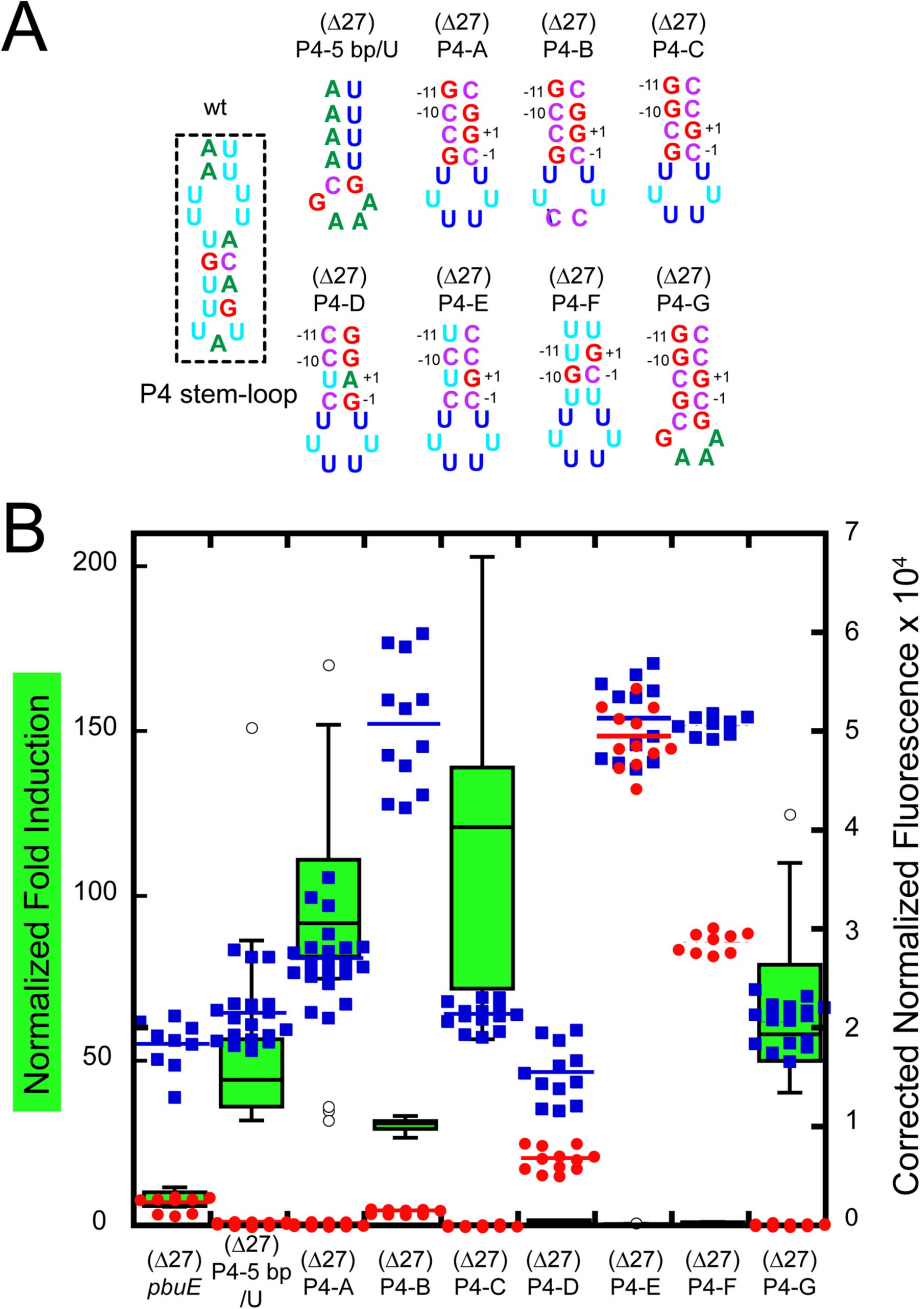
A consensus elemental pause and toehold within the P4 stem-loop is essential for regulatory switching. (A) Secondary structure of the P4 stem-loop variants; the aptamer domain remains unchanged. Numbering in variants (Δ27)P4-A through -G are the positions of the predicted elemental pause. (B) Normalized fold induction (green box-and-whisker plot) and background corrected normalized fluorescence data for each variant in the absence (red) and presence (blue) of 2AP. Format is that of previous figures.

To further define the contributions of specific sequence elements within the P4/L4 hairpin that contribute to regulatory activity, the P4-5 bp variant was examined in greater detail. First, to minimize potential effects from the pre-aptamer sequence, the first 27 nucleotides of the P4-5 bp/U riboswitch were deleted, yielding a riboswitch ((Δ27)P4-5 bp/U, Fig 7A) that has 45-fold induction of reporter expression in the presence of 2AP (Fig 7B). Secondly, to decouple the hexauridine tract from both P4 and P1, it was moved to the terminal loop ((Δ27)P4-A), which yielded a slightly more active variant than (Δ27)P4-5 bp (93-fold induction). However, this hexauridine tract, while important, when converted to a loop sequence of 5’UUCCUU yielded only a modest ~3-fold reduction in activity, indicating that it is not essential for 2AP-dependent regulation in the context of the P4 stem-loop variant “B”.

Instead, it was noticed that within P4 of variant (Δ27)P4-A is a potential “elemental pause sequence” (consensus is G_-11_,G_-10_/Y_-1_,G_+1_) that has been recently discovered via NET-seq approaches (Fig 7A) [57–59]. Conversion of this sequence (Fig 7A) to a strong consensus elemental pause in (Δ27)P4-C yields the highest fold induction (120-fold) of any variant tested in this study, while its elimination in (Δ27)P4-D causes complete loss of regulatory activity. Movement of the position of the elemental pause with in P4 weakens regulatory activity (S5 Fig) suggesting a context-dependence to its precise position but does not eliminate activity. Preservation of the elemental pause of (Δ27)P4-A but eliminating canonical base pairing P4 to disrupt the toehold ((Δ27)P4-D and -E) also destroys regulatory activity. Finally, conversion of the hexauridine loop of (Δ27)P4-C to a GAAA tetraloop in (Δ27)P4-G slightly reduces activity, but retains a high fold induction value. Of further note is that within this series most variants display a very low level of reporter expression in the absence of 2AP which is related to the very high level of induction seen in a subset of these variants. For some of these variants such as (Δ27)P4-A and (Δ27)P4-C, the levels of leaky reporter expression were reduced to near the background fluorescence observed in empty vector controls (S2 Table). Thus, the function of P4 is to provide both a pause site and a toehold in order to promote robust 2AP-dependent gene expression.

## Conclusions

**Fig 8:**
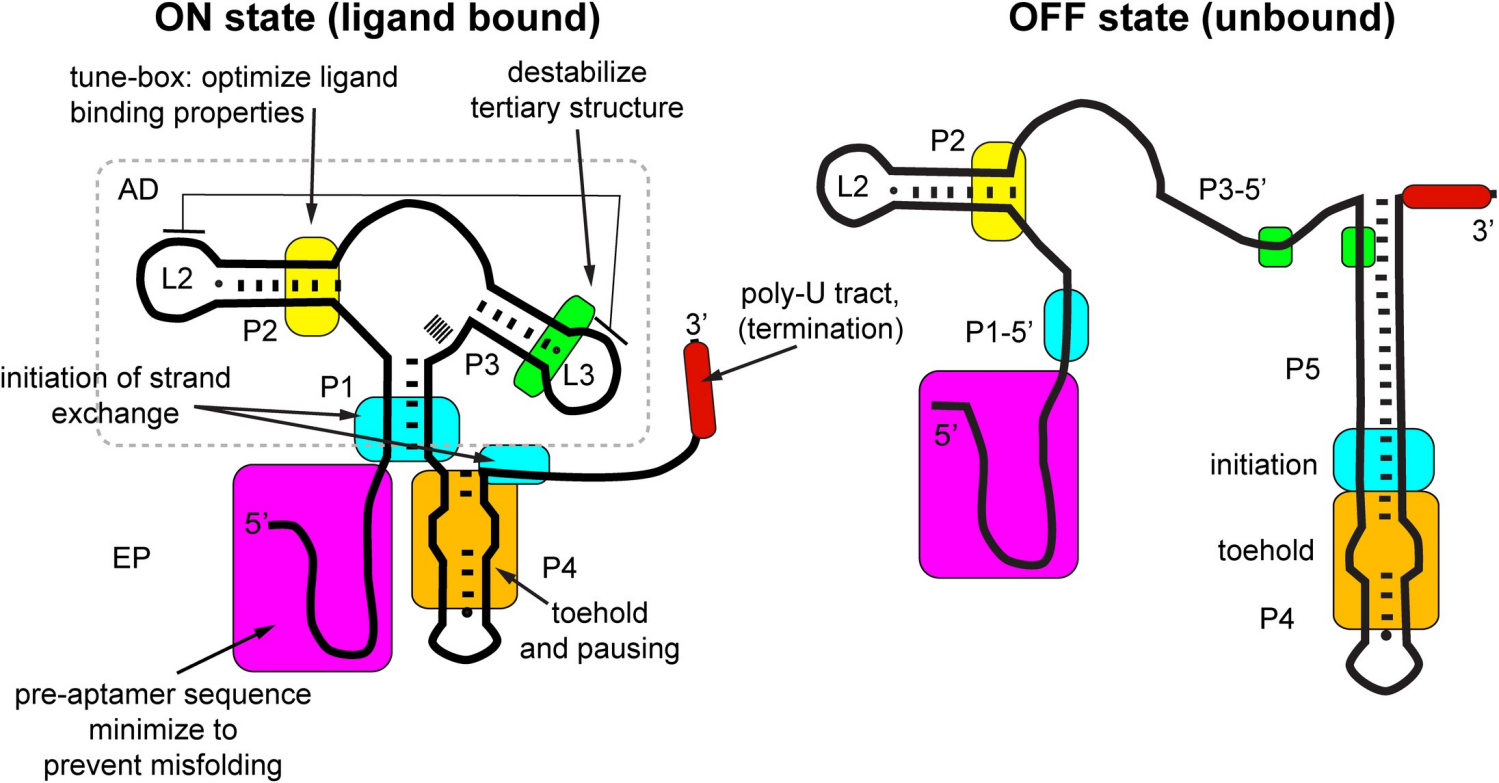
Map of functionally important features of the *pbuE* riboswitch. Cartoon representation of the *pbuE* riboswitch with colored boxes highlighting important regions for efficient regulatory activity. Note that the ligand binding site, the three-way junction, is omitted from this map to focus on components of the riboswitch not directly involved in ligand binding. The aptamer domain (AD) is boxed, with the remainder of the RNA considered to be the expression platform (EP). Positions of key elements are shown for both the ON (left) and OFF states (right).

In this study we have used a structure-guided mutagenesis strategy in concert with a cell-based fluorescent protein reporter assay to examine how sequence elements in both the aptamer and the expression platform influence the regulatory activity of a model transcriptional riboswitch. The mutagenesis strategy that we used, while revealing functional aspects of the wild type *B*. *subtilis pbuE* adenine riboswitch, principally focused upon understanding underlying principles of riboswitch function that should be considered in the design of novel riboswitches. Central to this approach was the substitution of key elements of the riboswitch—the aptamer domain and the P4 stem-loop of the expression platform—with similar elements and determining what is required to maximize the signal amplitude of 2AP-dependent regulation of reporter expression. Using this approach, we were able to unmask important aspects of the *pbuE* riboswitch likely not to have been revealed by a more limited mutagenesis strategy. Together, these data suggest a set of important considerations when designing functional riboswitches. Mapped onto the secondary structure of the ON state of the *pbuE* riboswitch (Fig 8), these elements are distributed throughout the RNA and affect different aspects of a ligand-gated strand exchange switching mechanism.

One of the most important design features of riboswitches is to avoid misfolded structures that lead to dysregulation. In the case of engineering the *pbuE* riboswitch, a combination of cell-based experiments and *in silico* modeling revealed that the single stranded pre-aptamer sequence (magenta, Fig 8), which is often ignored as part of the riboswitch, was problematic. In the context of the wild type *pbuE* riboswitch sequence this sequence has likely evolved to be neutral in the co-transcriptional folding process since its deletion had only small effects on the magnitude of the 2AP-mediated response. However, even small changes to the aptamer domain can result in misfolding involving the pre-aptamer sequence, similar to “RiboSNitches” in which single nucleotide variants dramatically alter the structure and function of an RNA [60,61]. Removal of the pre-aptamer sequence, as in the (Δ27) variants of this study, prevented this issue while not deleteriously impacting function. In general, given the rugged nature of a typical RNA’s folding landscape [62], elimination of all non-essential components of engineered riboswitches is likely an important path to optimizing activity.

Another design feature substantially improving performance is a programmed pause element. In natural riboswitches, it has long been recognized that transcriptional pauses occur within the expression platform to allow the aptamer domain more time to fold and bind ligand [13,54,55,63,64]. While the role of uridine tracts in riboswitch function has been explored [13,34,55], this study that has shown that a consensus elemental pause [57–59] appears to more strongly drive improvements in the dynamic range of the engineered *pbuE* riboswitch in *E*. *coli*. This is consistent with observations that in *B*. *subtilis* NusG promotes pausing at TTNTTT consensus sequences [65–67] while in *E*. *coli* NusG has the opposite effect by suppression of pause sites [68–70]. Interestingly, an elemental pause sequence located around the loop of the terminator was recently found to be an important constituent of the *Clostridium beijerinckii* ZTP riboswitch [50]. While in this study it was clear that either a hexauridine or elemental pause element improves the performance of the riboswitch, it should be noted that in several studies of pausing in *B*. *subtilis*, transcriptional pausing was not detected in the *pbuE* leader sequence [56,57]. Thus, while pausing within the *pbuE* riboswitch may not be important within its native genetic context, it is critical in a heterologous cellular context and thus should be considered an essential design feature.

A recent report revealed that the *B*. *subtilis* and *E*. *coli* RNA polymerases transcribe RNA at significantly differing rates that may help to further explain differences in pausing requirements [71]. Structural switching of the *pbuE* riboswitch may be optimized for decoupling of transcription and the pioneering round of translation, whereas in *E*. *coli* a strong pause is required to promote aptamer folding in the presence of a potentially interfering ribosome. Indeed, it is known that the *E*. *coli* ribosome can interfere with intrinsic terminator formation [72,73] whereas in *B*. *subtilis* this does not appear to be the case [71], further indicating that the co-transcriptional folding of the riboswitch may differ between the two organisms. Further, there are differences in the set of factors that influence transcription and its regulation between *E*. *coli* and *B*. *subtilis*; for example, *B*. *subtilis* lacks the Rho factor that is an important regulator of transcriptional termination in *E*. *coli*. Thus, design features of riboswitches not observed in wild type riboswitches may need to be considered to facilitate their function in certain bacteria such as *E*. *coli*. This question certainly requires more study to fully understand how pausing improves performance in the context of coupled transcription and translation.

Similar to changes in pausing, porting a riboswitch from its native genetic context to another organism may also result in differences in processing. Recent studies have revealed that an integral component of the mechanism of action of some riboswitches is processing and degradation of one conformation of the mRNA [74]. In *E*. *coli*, many native riboswitches use a dual regulatory mechanism involving translational initiation via exposure or occlusion of the ribosome binding site and some other aspect of RNA processing by indirectly or directly modulating Rho-factor or RNase E accessibility [75–77]. Further, a cobalamin riboswitch that was ported from an organism in a marine metagenome to *E*. *coli* appears to operate by dual control of translational initiation and mRNA stability [78]. In *B*. *subtilis*, RNase Y—functionally similar to RNase E in *E*. *coli*—has been shown to be important for processing the *yitJ* SAM-I riboswitch and likely other riboswitches as well [79,80]. Another RNA processing enzyme, RNase P, has also been suggested to play a role in processing riboswitches, including *pbuE* [81,82]. In the current study, we have not attempted to address this potential component of regulation in our designs, but how designed riboswitches are affected by RNA processing may be an important component for further performance optimization.

A third important design feature of a ligand-gated transcriptional riboswitch is efficient formation of alternative secondary structure via strand exchange. In the *pbuE* riboswitch, there are three regions of the aptamer domain and expression platform that significantly affect this process. The first, unmasked by *xpt*/pbuE* chimeric riboswitch, is to have tertiary structure compatible with strand exchange. In *xpt*, the L2-L3 interaction is highly stable in the absence of ligand [24–26], preventing full strand exchange and formation of the base of the terminator hairpin—an essential component of the intrinsic terminator [83]. Destabilization of this interaction by the introduction of a non-canonical A-A pair at the base of L3 (green, Fig 8) alleviates this block and enables full strand exchange. This indicates that the relationship between ligand-independent tertiary structure in the aptamer domain and alternative secondary structure must be carefully considered. Of course, ligand binding to the three-way junction also induces tertiary structure in the aptamer that is intended to block strand exchange highlighting the importance of higher-order structure in controlling the exchange process.

Another essential feature of strand exchange is the efficient nucleation of the event using a toehold sequence and the site of initiation of strand invasion (orange, Fig 8). Toehold sequences are critical design elements in the design of a variety of nucleic acids devices including biosensors and riboregulators [84,85]. In natural RNAs, the presence of a toehold to efficiently nucleate strand exchange have also been observed [86]. In addition to the toehold, sequences within the strand exchange may have a significant contribution to the efficiency of this process, as recently observed in the ZTP riboswitch [50]. Sequences at the site of initiation of strand invasion—the three base pairs of P1 that are distal to the junction—also have a significant impact upon the efficiency of switching. In the absence of ligand, sequences that have weak base pairs both in P1 and the invader ((Δ27)P1-AU, -GU; Fig 4D) are unable to efficiently form the terminator and exhibit high amounts of reporter fluorescence, whereas variants dominated by strong base pairs in both P1 and the invader are strongly terminated in the absence of ligand ((Δ27)P1-GC2b, -GC3) indicating that invasion proceeds efficiently through the P1 helix. These results suggest that tuning the strength of base pairing in competing secondary structure can enable tuning of the dynamic range of the switch. In the case of *pbuE*, optimal performance occurs with a single G-C base pair as in wild type, or two G-C pairs separated by a weaker pair. This indicates that while the process of stand exchange can be rapid, the timeframe between initiation of strand exchange and the decision point at the intrinsic terminator may be sufficiently short that the sequence composition of the exchanging structures still significantly influences performance. This is consistent with single molecule studies of spontaneous branch migration of DNA Holliday junctions which suggest a rugged energy landscape with sequence-dependent barriers [87].

Alteration and optimizing features of the *pbuE* riboswitch expression platform yielded a variant ((Δ27)P4-C) that has significantly improved signal amplitude over wild type (122-fold and 5.6-fold, respectively). This improvement in the signal amplitude is mostly due to suppression of read through transcription in the absence of ligand, which is mostly the result of elimination of the pre-aptamer sequence, a strong toehold and an already near-optimal sequence in P1 in the wild type *pbuE* aptamer to promote strand exchange. Further studies will be required to determine if these changes also yield improvements in sensitivity. This will require titrations of these variants with 2AP to measure the concentration of ligand required to elicit a half-maximal regulatory response both *in vitro* (T_50_) and in the cellular environment (E_50_).

The ability to rationally engineer synthetic riboswitches that function robustly in cells and *in vitro* cells open doors for many applications. For example, chimeric riboswitches based upon aptamer/expression platform “mix-and-match” strategies have been successfully implemented in the cyanobacterium *Anabaena* sp. PCC 7120 [88] and in *Synechococcus elongatus* [89], both proposed to be model organisms for photosynthetic production of useful chemicals. Further, a modified *pbuE* riboswitch was recently shown to be able to robust control gene expression in the moderate thermophiles *Geobacillus thermoglucosidasius* and *Clostridium thermocellum*, suggesting that these devices maybe useful in organisms that can grow under higher fermentation temperatures that is desired for the industrial production of some chemicals [90]. With respect to *in vitro* applications, riboswitches as field-deployable or point-of-care biosensors that detect a specific small molecule and provide a rapid visual readout are becoming increasingly practical [11]. For example, the fluoride riboswitch that controls expression of a downstream fluorophore-binding aptamer was developed into a field-deployable sensor that can rapidly assess fluoride ion concentrations in municipal water supplies with sufficient sensitivity to detect concentrations that exceed acceptable levels by only a few fold [91]. More recently, a Cas13-based approach has been developed that can covert detection of a small molecule by a riboswitch into a fluorescence output in a single-pot, isothermal reaction in ten minutes [92]. The bottleneck in extending these applications to a greater variety of small molecule sensors is the development novel biosensors with efficient coupling of an input (the aptamer) to a readily-detectible output.

## Supporting information

S1 Fig Representative growth curves of BW25113 cells transformed with select riboswitch variant containing reporter vectors. Growths were performed in CSB medium at 37°C in the absence (red circles) or presence (blue squares) of 1 mM 2-aminopurine. (A) BW25113 transformed with pBR322. (B) BW25113 transformed with (Δ27) *pbuE*. (C) BW25113 transformed with (Δ27) P4-5 bp/U. (D) BW25113 transformed with (Δ27) P4-A. Variants in panels B, C and D are associated with Fig 7. Note that all assays in this work were performed with cells grown to 0.3–0.5 O.D. (600 nm).
(DOCX)

S2 Fig Engineering of the pre-aptamer sequence of *pbuE*. Direct comparison of wild type (wt) *pbuE*, *pbuE* with first 11 nucleotides removed from the pre- aptamer sequence (Δ11), and *pbuE* with 11 nucleotides removed and additional *Aat*II and *Spe*I restriction sites added (Δ11,RS) and *pbuE* with the first 27 nucleotides removed (Δ27). Each control was assayed in the absence (red) and presence (blue) of 2AP with the fold induction reported in a standard boxplot format in green. Red and blue bars represent the median value and the dashed line represents a fold induction value of 1 (no induction of reporter expression in the presence of 2AP).
(DOCX)

S3 Fig Structure of *purE*/pbuE* hybrid with highlighted repair mutations. Nucleotide coloring scheme is the same as Fig 1. Pre-aptamer leader sequence removed for simplicity but is the same as pbuE*.
(DOCX)

S4 Fig Structure of *yxjA*/pbuE* chimera with highlighted repair mutations. Nucleotide coloring scheme is the same as for Fig 1. Pre-aptamer leader sequence removed for simplicity, but is the same as pbuE*.
(DOCX)

S5 Fig Riboswitch variants in which the placement of the elemental pause has been shifted within the helix of the P4 stem-loop. (A) Secondary structure of the P4 stem-loop for each variant with the position of the elemental pause denoted. (B) (B) Normalized fold induction and background corrected normalized fluorescence data for each variant in the absence (red) and presence (blue) of 2AP.
(DOCX)

S1 Table Riboswitch variant sequences. ^a^Full promoter and leader of parental *pbuE* sequences (S1 Table) through the initiator ATG codon of the reporter gene. The promoter is underlined and italicized, the pre-aptamer sequence is in bold. ^b^Full leader sequence in bold with mutations underlined of each of the remaining riboswitch mutants.
(DOCX)

S2 Table Data values for Figs 2–7 and S1 Fig. ^a^Median vaule of background normalized fluorescence with each measurement represents at least three technical replicates of three independent biological replicates. Errors are reported as standard errors as calculated in Excel.
(DOCX)
