# Supplementary material for: Requirements for efficient ligand-gated co-transcriptional switching in designed variants of the B. subtilis pbuE adenine-responsive riboswitch in E. coli
Source: PLoS One. 2020 Dec 1;15(12):e0243155. doi: 10.1371/journal.pone.0243155 (PMC7707468; doi:10.1371/journal.pone.0243155)
Supplement: S1 Fig — Growths were performed in CSB medium at 37°C in the absence (red circles) or presence (blue squares) of 1 mM 2-aminopurine. (A) BW25113 transformed with pBR322. (B) BW25113 transformed with (Δ27) pbuE. (C) BW25113 transformed with (Δ27) P4-5 bp/U. (D) BW25113 transformed with (Δ27) P4-A. Variants in panels B, C and D are associated with Fig 7. Note that all assays in this work were performed with cells grown to 0.3–0.5 O.D. (600 nm). (DOCX) [file pone.0243155.s001.docx]

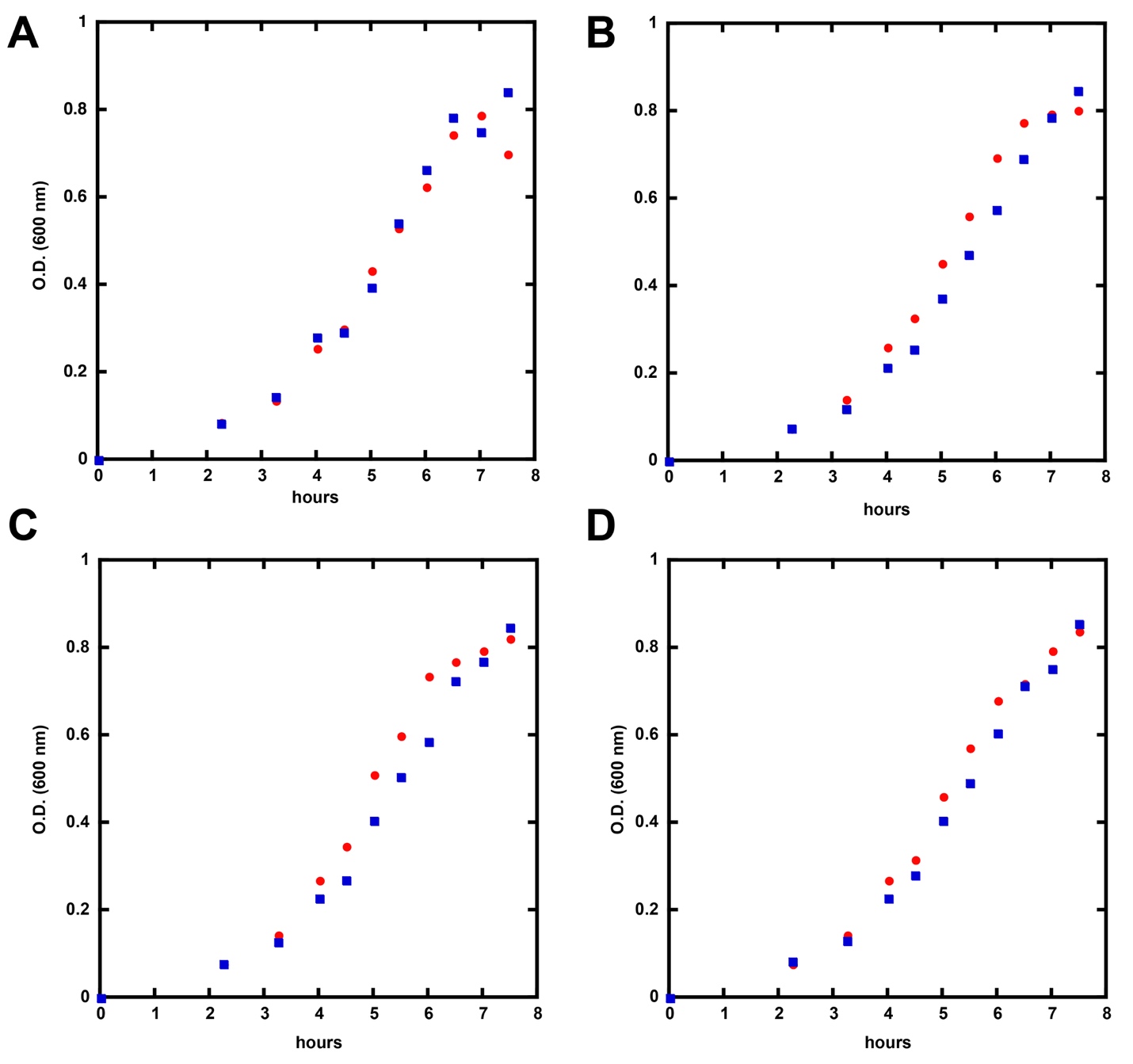


**S1 Figure. Representative growth curves of BW25113 cells transformed with select riboswitch variant containing reporter vectors.** Growths were performed in CSB medium at 37 °C in the absence (red circles) or presence (blue squares) of 1 mM 2-aminopurine. (A) BW25113 transformed with pBR322. (B) BW25113 transformed with (∆27) *pbuE.* (C) BW25113 transformed with (∆27) P4-5 bp/U. (D) BW25113 transformed with (∆27) P4-A. Variants in panels B, C and D are associated with Figure 7. Note that all assays in this work were performed with cells grown to 0.3 – 0.5 O.D. (600 nm).
