# Supplementary material for: Requirements for efficient ligand-gated co-transcriptional switching in designed variants of the B. subtilis pbuE adenine-responsive riboswitch in E. coli
Source: PLoS One. 2020 Dec 1;15(12):e0243155. doi: 10.1371/journal.pone.0243155 (PMC7707468; doi:10.1371/journal.pone.0243155)
Supplement: S2 Fig — Direct comparison of wild type (wt) pbuE, pbuE with first 11 nucleotides removed from the pre- aptamer sequence (Δ11), and pbuE with 11 nucleotides removed and additional AatII and SpeI restriction sites added (Δ11,RS) and pbuE with the first 27 nucleotides removed (Δ27). Each control was assayed in the absence (red) and presence (blue) of 2AP with the fold induction reported in a standard boxplot format in green. Red and blue bars represent the median value and the dashed line represents a fold induction value of 1 (no induction of reporter expression in the presence of 2AP). (DOCX) [file pone.0243155.s002.docx]

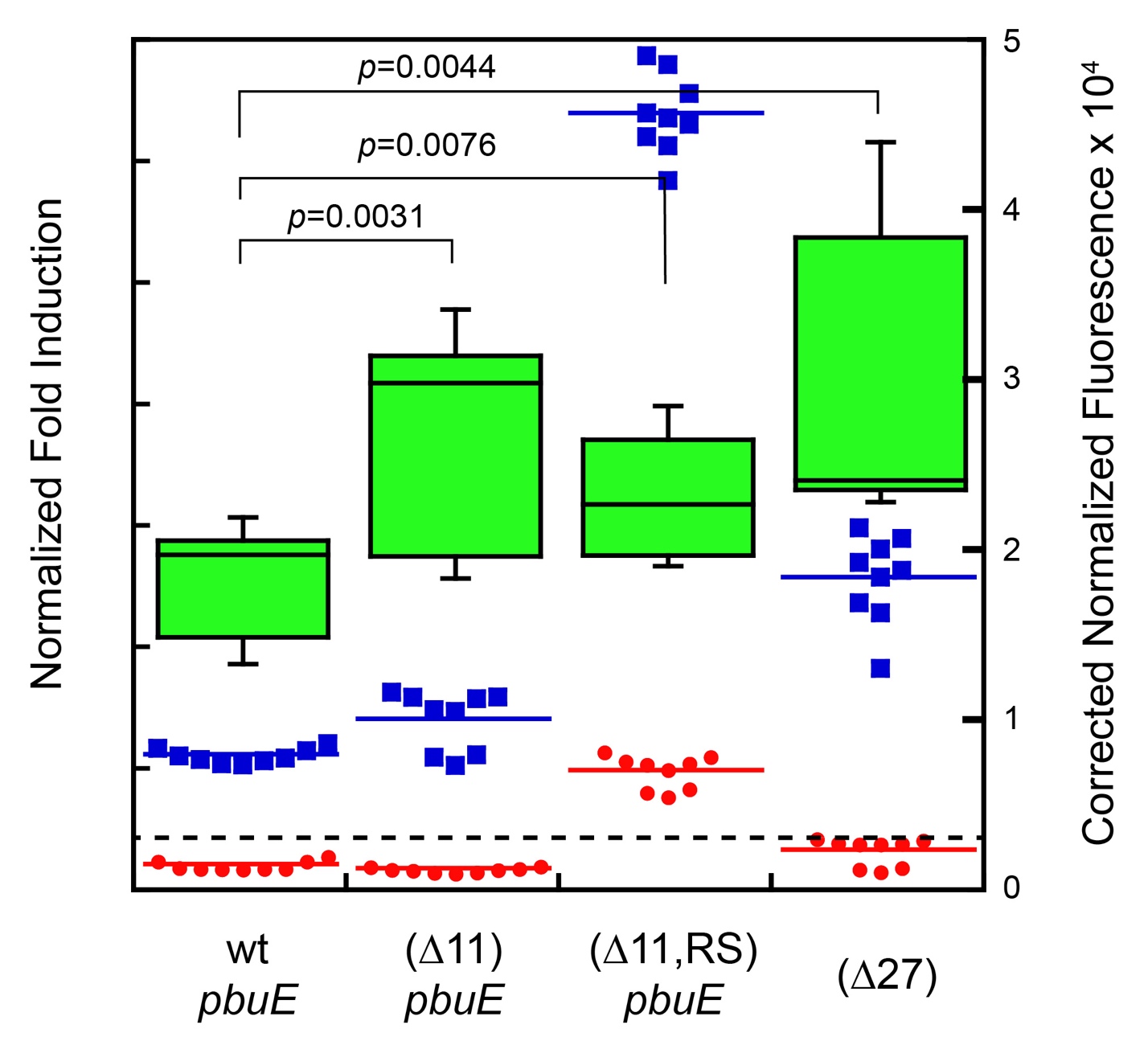


**S2 Figure. Engineering of the pre-aptamer sequence of *pbuE*.** Direct comparison of wild type (wt) *pbuE*, *pbuE* with first 11 nucleotides removed from the pre- aptamer sequence (∆11), and *pbuE* with 11 nucleotides removed and additional *Aat*II and *Spe*I restriction sites added (∆11,RS) and *pbuE* with the first 27 nucleotides removed (∆27). Each control was assayed in the absence (red) and presence (blue) of 2AP with the fold induction reported in a standard boxplot format in green. Red and blue bars represent the median value and the dashed line represents a fold induction value of 1 (no induction of reporter expression in the presence of 2AP).
