# Supplementary material for: Requirements for efficient ligand-gated co-transcriptional switching in designed variants of the B. subtilis pbuE adenine-responsive riboswitch in E. coli
Source: PLoS One. 2020 Dec 1;15(12):e0243155. doi: 10.1371/journal.pone.0243155 (PMC7707468; doi:10.1371/journal.pone.0243155)
Supplement: S4 Fig — Nucleotide coloring scheme is the same as for Fig 1. Pre-aptamer leader sequence removed for simplicity, but is the same as pbuE*. (DOCX) [file pone.0243155.s004.docx]

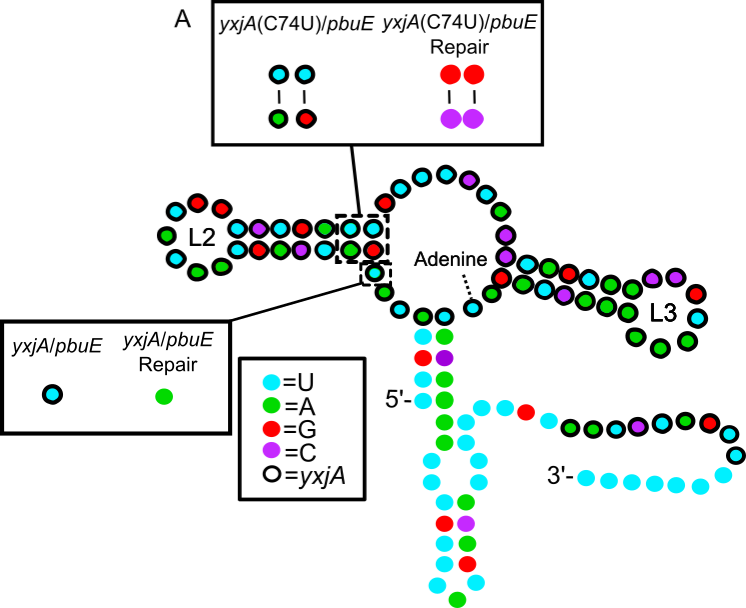


**S4 Figure. Structure of *yxjA*/*pbuE** chimera with highlighted repair mutations.** Nucleotide coloring scheme is the same as for Fig 1. Pre-aptamer leader sequence removed for simplicity, but is the same as *pbuE**.
