# Supplementary material for: Requirements for efficient ligand-gated co-transcriptional switching in designed variants of the B. subtilis pbuE adenine-responsive riboswitch in E. coli
Source: PLoS One. 2020 Dec 1;15(12):e0243155. doi: 10.1371/journal.pone.0243155 (PMC7707468; doi:10.1371/journal.pone.0243155)
Supplement: S5 Fig — (A) Secondary structure of the P4 stem-loop for each variant with the position of the elemental pause denoted. (B) (B) Normalized fold induction and background corrected normalized fluorescence data for each variant in the absence (red) and presence (blue) of 2AP. (DOCX) [file pone.0243155.s005.docx]

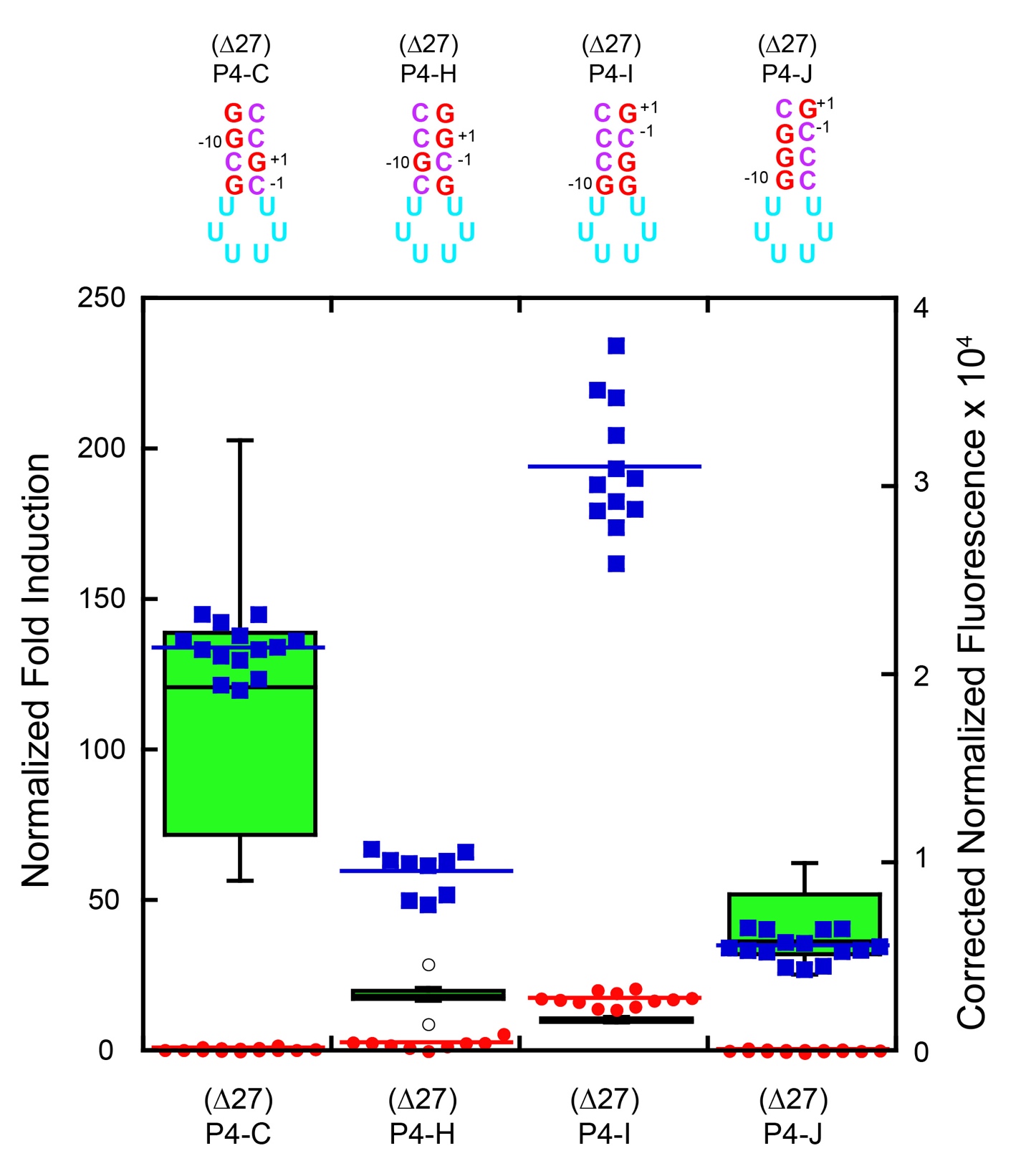


**S5 Figure. Riboswitch variants in which the placement of the elemental pause has been shifted within the helix of the P4 stem-loop.** (A) Secondary structure of the P4 stem-loop for each variant with the position of the elemental pause denoted. (B) (B) Normalized fold induction and background corrected normalized fluorescence data for each variant in the absence (red) and presence (blue) of 2AP.
