# Supplementary material for: Requirements for efficient ligand-gated co-transcriptional switching in designed variants of the B. subtilis pbuE adenine-responsive riboswitch in E. coli
Source: PLoS One. 2020 Dec 1;15(12):e0243155. doi: 10.1371/journal.pone.0243155 (PMC7707468; doi:10.1371/journal.pone.0243155)
Supplement: S1 Table — aFull promoter and leader of parental pbuE sequences (S1 Table) through the initiator ATG codon of the reporter gene. The promoter is underlined and italicized, the pre-aptamer sequence is in bold. bFull leader sequence in bold with mutations underlined of each of the remaining riboswitch mutants. (DOCX) [file pone.0243155.s006.docx]

| Riboswitch variants | Sequence (DNA) |
| --- | --- |
| pbuE variants |  |
| wild type *pbuE*^a^ | TTTACGGGCATGCATAAGGCTCGTATAATATATTC**GGAAACGAATCAATTAAATAGCTATTATCAC**TTGTATAACCTCAATAATATGGTTTGAGGGTGTCTACCAGGAACCGTAAAATCCTGATTACAAAATTTGTTTATGACATTTTTTGTAATCAGGATTTTTTTTATTTATCAAAACATTTAAGTAAAGGAGTTTGTTATG |
| ∆11/RS *pbuE*^a^  (*pbuE**) | *TTTACGGGCATGCATAAGGCTCGTATAATATATTC***AATTAAATAGACGTCATTATCAC**TTGTATAACCTCAATAATATGGTTTGAGGGTGTCTACCAGGAACCGTAAAATCCTGATTACAAAATTTGTTTATGACATTTTTTGTAATCAGGATTTTTTTTATTTACTAGTACATTTAAGTAAAGGAGTTATG |
| Δ11 *pbuE* | *TTTACGGGCATGCATAAGGCTCGTATAATATATTC***AATTAAATAGCTATTATCAC**TTGTATAACCTCAATAATATGGTTTGAGGGTGTCTACCAGGAACCGTAAAATCCTGATTACAAAATTTGTTTATGACATTTTTTGTAATCAGGATTTTTTTTATTTATCAAAACATTTAAGTAAAGGAGTTTGTTATG |
| ∆27 *pbuE* | TTTACGGGCATGCATAAGGCTCGTATAATATATTC**CAC**TTGTATAACCTCAATAATATGGTTTGAGGGTGTCTACCAGGAACCGTAAAATCCTGATTACAAAATTTGTTTATGACATTTTTTGTAATCAGGATTTTTTTTATTTACTAGTACATTTAAGTAAAGGAGTTATG |
| *pbuE* expression platform modularity |  |
| *xpt*(C74U)/*pbuE** (A)^b^ | **AATTAAATAGACGTCATTATCAC**TTGTATAATCGCGTGGATATGGCACGCAAGTTTCTACCGGGCACCGTAAATGTCCGATTACAAAATTTGTTTATGACATTTTTTGTAATCAGGATTTTTTTT |
| *xpt(*C74U)/*pbuE** A-A P3 (B) | **AATTAAATAGACGTCATTATCAC**TTGTATAATCGCGTGGATATGGCACGCAAGTTTCTACCGGGCACCGTAAA**A**GTCCGATTACAAAATTTGTTTATGACATTTTTTGTAATCAGGATTTTTTTT |
| *xpt*(C74U)/*pbuE** 2GC Tunebox (C) | **AATTAAATAGACGTCATTATCAC**TTGTATAA**CC**GCGTGGATATGGCACGCGGGTTTCTACCGGGCACCGTAAATGTCCGATTACAAAATTTGTTTATGACATTTTTTGTAATCAGGATTTTTTTT |
| *xpt*(C74U)/*pbuE** A-A P3 2GC Tunebox (D) | **AATTAAATAGACGTCATTATCAC**TTGTATAA**CC**GCGTGGATATGGCACGCGGGTTTCTACCGGGCACCGTAAAAGTCCGATTACAAAATTTGTTTATGACATTTTTTGTAATCAGGATTTTTTTT |
| *xpt*(C74U)/*pbuE** A-A P3 Paired Tunebox (E) | **AATTAAATAGACGTCATTATCAC**TTGTATAATCGCGTGGATATGGCACGCGAGTTTCTACCGGGCACCGTAAAAGTCCGATTACAAAATTTGTTTATGACATTTTTTGTAATCAGGATTTTTTTT |
| *yxjA/pbuE** hybrid | **AATTAAATAGACGTCATTATCAC**TTGTATATGATCAGTAATATGGTCTGATTGTTTCTACCTAGTAACCGTAAAAAACTAGATTACAAAATTTGTTTATGACATTTTTTGTAATCTAGTTTTTTT |
| *yxjA/pbuE** repair hybrid | **AATTAAATAGACGTCATTATCAC**TTGTATAACCTCAGTAATATGGTCTGAGGGTTTCTACCTAGTAACCGTAAAAAACTAGATTACAAAATTTGTTTATGACATTTTTTGTAATCTAGTTTTTTT |
| *purE/pbuE** hybrid | **AATTAAATAGACGTCATTATCAC**TTGTATAATCTTGGGAATATGGCCCATAAGTTTCTACCCGGCAACCGTAAATTGCCGGATTACAAAATTTGTTTATGACATTTTTTGTAATCCGGCAATTTT |
| *purE/pbuE** repair hybrid | **AATTAAATAGACGTCATTATCAC**TTGTATAATCTTGGGAATATGGCCCAAGAGTTTCTACCCGGCAACCGTAAAATGCCGGATTACAAAATTTGTTTATGACATTTTTTGTAATCCGGCATTTTT |
| P1 helix mutants |  |
| P1-AU | **AATTAAATAGACGTCATTATCAC**TTATATAACCTCAATAATATGGTTTGAGGGTGTCTACCAGGAACCGTAAAATCCTGATTATAAAATTTGTTTATGACATTTTTTATAATCAGGATTTTTTTT |
| P1-GU | **AATTAAATAGACGTCATTATCAC**TTGTATAACCTCAATAATATGGTTTGAGGGTGTCTACCAGGAACCGTAAAATCCTGATTATAAAATTTGTTTATGACATTTTTTATAATCAGGATTTTTTTT |
| P1-GC2a | **AATTAAATAGACGTCATTATCAC**TCGTATAACCTCAATAATATGGTTTGAGGGTGTCTACCAGGAACCGTAAAATCCTGATTACGAAATTTGTTTATGACATTTTTCGTAATCAGGATTTTTTTT |
| P1-GC2b | **AATTAAATAGACGTCATTATCAC**CTGTATAACCTCAATAATATGGTTTGAGGGTGTCTACCAGGAACCGTAAAATCCTGATTACAGAATTTGTTTATGACATTTTCTGTAATCAGGATTTTTTTT |
| P1-GC3 | **AATTAAATAGACGTCATTATCAC**CCGTATAACCTCAATAATATGGTTTGAGGGTGTCTACCAGGAACCGTAAAATCCTGATTACGGAATTTGTTTATGACATTTTCCGTAATCAGGATTTTTTTT |
| (∆27) P1-AU | **CAC**TTATATAACCTCAATAATATGGTTTGAGGGTGTCTACCAGGAACCGTAAAATCCTGATTATAAAATTTGTTTATGACATTTTTTATAATCAGGATTTTTTTT |
| (∆27) P1-GU | **CAC**TTGTATAACCTCAATAATATGGTTTGAGGGTGTCTACCAGGAACCGTAAAATCCTGATTATAAAATTTGTTTATGACATTTTTTATAATCAGGATTTTTTTT |
| (∆27) P1-GC2a | **CAC**TCGTATAACCTCAATAATATGGTTTGAGGGTGTCTACCAGGAACCGTAAAATCCTGATTACGAAATTTGTTTATGACATTTTTCGTAATCAGGATTTTTTTT |
| (∆27) P1-GC2b | **CAC**CTGTATAACCTCAATAATATGGTTTGAGGGTGTCTACCAGGAACCGTAAAATCCTGATTACAGAATTTGTTTATGACATTTTCTGTAATCAGGATTTTTTTT |
| (∆27) P1-GC3 | **CAC**CCGTATAACCTCAATAATATGGTTTGAGGGTGTCTACCAGGAACCGTAAAATCCTGATTACGGAATTTGTTTATGACATTTTCCGTAATCAGGATTTTTTTT |
| Misfolding Repair Variants |  |
| P1-GC2b Repair | **AATTAAATTGACGTCATTAT**ACTGTATAACCTCAATAATATGGTTTGAGGGTGTCTACCAGGAACCGTAAAATCCTGATTACAGAATTTGTTTATGACATTTTCTGTAATCAGGATTTTTTTT |
| P1-GC3 Repair | **AATTAAAATGACGTCATTACT**CCGTATAACCTCAATAATATGGTTTGAGGGTGTCTACCAGGAACCGTAAAATCCTGATTACGGAATTTGTTTATGACATTTTCCGTAATCAGGATTTTTTTT |
| Synthetic terminator helices |  |
| P4-0 bp-0 | **AATTAAATAGACGTCATTATCAC**TTGTATAACCTCAATAATATGGTTTGAGGGTGTCTACCAGGAACCGTAAAATCCTGATTACAAGAAATTGTAATCAGGATTTTTTTT |
| P4-0 bp/U | **AATTAAATAGACGTCATTATCAC**TTGTATAACCTCAATAATATGGTTTGAGGGTGTCTACCAGGAACCGTAAAATCCTGATTACAATTTTTTGTAATCAGGATTTTTTTT |
| P4-2 bp | **AATTAAATAGACGTCATTATCAC**TTGTATAACCTCAATAATATGGTTTGAGGGTGTCTACCAGGAACCGTAAAATCCTGATTACAACCGAAAGGTTGTAATCAGGATTTTTTTT |
| P4-4 bp | **AATTAAATAGACGTCATTATCAC**TTGTATAACCTCAATAATATGGTTTGAGGGTGTCTACCAGGAACCGTAAAATCCTGATTACAAAGGCGAAAGCCTTTGTAATCAGGATTTTTTTT |
| P4-4 bp/U | **AATTAAATAGACGTCATTAT**CACTTGTATAACCTCAATAATATGGTTTGAGGGTGTCTACCAGGAACCGTAAAATCCTGATTACAAAAAAGAAATTTTTTGTAATCAGGATTTTTTTT |
| P4-5 bp | **AATTAAATAGACGTCATTATCAC**TTGTATAACCTCAATAATATGGTTTGAGGGTGTCTACCAGGAACCGTAAAATCCTGATTACAACCAACGAAAGTTGGTTGTAATCAGGATTTTTTTT |
| P4-5 bp/U | **AATTAAATAGACGTCATTATCAC**TTGTATAACCTCAATAATATGGTTTGAGGGTGTCTACCAGGAACCGTAAAATCCTGATTACAAAAAACGAAAGTTTTTTGTAATCAGGATTTTTTTT |
| NH-5 6U Loop | **AATTAAATAGACGTCATTATCAC**TTGTATAACCTCAATAATATGGTTTGAGGGTGTCTACCAGGAACCGTAAAATCCTGATTACAAGCCGTTTTTTCGGCTTGTAATCAGGATTTTTTTT |
| P4-6 bp | **AATTAAATAGACGTCATTATCAC**TTGTATAACCTCAATAATATGGTTTGAGGGTGTCTACCAGGAACCGTAAAATCCTGATTACAAAATGGCGAAAGCCATTTTGTAATCAGGATTTTTTTT |
| P4-8 bp | **AATTAAATAGACGTCATTATCAC**TTGTATAACCTCAATAATATGGTTTGAGGGTGTCTACCAGGAACCGTAAAATCCTGATTACAAAAAACGGCGAAAGCCGTTTTTTGTAATCAGGATTTTTTTT |
| P4-10 bp | **AATTAAATAGACGTCATTATCAC**TTGTATAACCTCAATAATATGGTTTGAGGGTGTCTACCAGGAACCGTAAAATCCTGATTACAAAAAACCTGGCGAAAGCCAGGTTTTTTGTAATCAGGATTTTTTTT |
| P4-5 bp variants |  |
| (∆27) P4-5 bp/U | **CAC**TTGTATAACCTCAATAATATGGTTTGAGGGTGTCTACCAGGAACCGTAAAATCCTGATTACAAAAAACGAAAGTTTTTTGTAATCAGGATTTTTTTT |
| (∆27) P4-A | **CAC**TTGTATAACCTCAATAATATGGTTTGAGGGTGTCTACCAGGAACCGTAAAATCCTGATTACAAGCCGTTTTTTCGGCTTGTAATCAGGATTTTTTTT |
| (∆27) P4-B | **CAC**TTGTATAACCTCAATAATATGGTTTGAGGGTGTCTACCAGGAACCGTAAAATCCTGATTACAAGCCGTTCCTTCGGCTTGTAATCAGGATTTTTTTT |
| (∆27) P4-C | **CAC**TTGTATAACCTCAATAATATGGTTTGAGGGTGTCTACCAGGAACCGTAAAATCCTGATTACAAGGCGTTTTTTCGCCTTGTAATCAGGATTTTTTTT |
| (∆27) P4-D | **CAC**TTGTATAACCTCAATAATATGGTTTGAGGGTGTCTACCAGGAACCGTAAAATCCTGATTACAACCTCTTTTTTGAGGTTGTAATCAGGATTTTTTTT |
| (∆27) P4-E | **CAC**TTGTATAACCTCAATAATATGGTTTGAGGGTGTCTACCAGGAACCGTAAAATCCTGATTACAATCTCTTTTTTCGCCTTGTAATCAGGATTTTTTTT |
| (∆27) P4-F | **CAC**TTGTATAACCTCAATAATATGGTTTGAGGGTGTCTACCAGGAACCGTAAAATCCTGATTACAATTGTTTTTTTTCGTTTGTAATCAGGATTTTTTTT |
| (∆27) P4-G | **CAC**TTGTATAACCTCAATAATATGGTTTGAGGGTGTCTACCAGGAACCGTAAAATCCTGATTACAAGGCGCGAAAGCGCCTTGTAATCAGGATTTTTTTT |
|  |  |

**S1 Table. Riboswitch variant sequences***.* ^a^Full promoter and leader of parental *pbuE* sequences (Table S1) through the initiator ATG codon of the reporter gene. The promoter is underlined and italicized, the pre-aptamer sequence is in bold. ^b^Full leader sequence in bold with mutations underlined of each of the remaining riboswitch mutants.
