# Supplementary material for: Requirements for efficient ligand-gated co-transcriptional switching in designed variants of the B. subtilis pbuE adenine-responsive riboswitch in E. coli
Source: PLoS One. 2020 Dec 1;15(12):e0243155. doi: 10.1371/journal.pone.0243155 (PMC7707468; doi:10.1371/journal.pone.0243155)
Supplement: S2 Table — aMedian vaule of background normalized fluorescence with each measurement represents at least three technical replicates of three independent biological replicates. Errors are reported as standard errors as calculated in Excel. (DOCX) [file pone.0243155.s007.docx]

| Riboswitch Variant | Median Normalized Fluorescence, no 2AP^a^  (arbitrary units, A.U.) | Median Normalized Fluorescence, 1 mM 2AP  (arbitrary units, A.U.) | Median Fold Induction |
| --- | --- | --- | --- |
| pbuE variants |  |  |  |
| wild type *pbuE*^a^ | 1420±90 | 7930±140 | 5.6±0.3 |
| ∆11/RS *pbuE*^a^  (*pbuE**) | 1350±50 | 10800±900 | 8.4±0.6 |
| Δ11 *pbuE* | 7540±320 | 45600±800 | 6.4±0.3 |
| ∆27 *pbuE* | 2850±270 | 19000±900 | 6.8±0.8 |
| *pbuE* expression platform modularity |  |  |  |
| *xpt*(C74U)/*pbuE** (A)^b^ | 18800±1200 | 28100±2100 | 1.8±0.2 |
| *xpt(*C74U)/*pbuE** A-A P3 (B) | 5420±350 | 9220±1400 | 1.9±0.2 |
| *xpt*(C74U)/*pbuE** 2GC Tunebox (C) | 25200±1400 | 45900±5200 | 2.0±0.2 |
| *xpt*(C74U)/*pbuE** A-A P3 2GC Tunebox (D) | 8380±510 | 55100±1900 | 6.5±0.3 |
| *xpt*(C74U)/*pbuE** A-A P3 Paired Tunebox (E) | 7940±190 | 57400±1100 | 7.5±0.2 |
| *yxjA/pbuE** hybrid | 2320±170 | 2690±210 | 1.2±0.1 |
| *yxjA/pbuE** repair hybrid | 1250±40 | 5110±150 | 4.2±0.2 |
| *purE/pbuE** hybrid | 1150±110 | 1230±130 | 1.4±0.1 |
| *purE/pbuE** repair hybrid | 1290±20 | 3200±30 | 2.5±0.1 |
| P1 helix mutants |  |  |  |
| P1-AU | 25300±400 | 89100±1900 | 3.3±0.1 |
| P1-GU | 32500±500 | 87300±3700 | 2.7±0.1 |
| P1-GC2a | 19600±700 | 87500±4700 | 4.4±0.1 |
| P1-GC2b | 11800±700 | 35600±2000 | 2.7±0.1 |
| P1-GC3 | 9720±700 | 16500±1500 | 1.5±0.1 |
| (∆27) P1-AU | 24900±900 | 23900±1100 | 1.0±0.1 |
| (∆27) P1-GU | 4290±80 | 7540±350 | 1.8±0.1 |
| (∆27) P1-GC2a | 1730±80 | 7680±600 | 5.6±0.2 |
| (∆27) P1-GC2b | 1290±50 | 13300±300 | 9.6±0.5 |
| (∆27) P1-GC3 | 1290±50 | 2270±110 | 1.6±0.1 |
| Misfolding Repair Variants |  |  |  |
| P1-GC2b Repair | 2510±60 | 15500±500 | 6.4±0.3 |
| P1-GC3 Repair | 330±70 | 320±60 | 1.1±0.4 |
| Synthetic terminator helices |  |  |  |
| P4-0 bp-0 | 27100±600 | 63400±5000 | 2.3±0.2 |
| P4-0 bp/U | 5690±320 | 21500±690 | 3.5±0.3 |
| P4-2 bp | 19400±300 | 44200±1400 | 2.3±0.1 |
| P4-4 bp | 7730±410 | 24400±1600 | 3.7±0.2 |
| P4-4 bp/U | 5310±290 | 44400±2800 | 9.0±0.3 |
| P4-5 bp | 4190±240 | 6250±380 | 1.5±0.1 |
| P4-5 bp/U | 980±20 | 8810±110 | 9.1±0.2 |
| P4-6 bp | 32200±560 | 142000±4000 | 4.3±0.1 |
| P4-8 bp | 1760±190 | 28100±1400 | 15±1 |
| P4-10 bp | 1820±110 | 13800±600 | 7.1±0.7 |
| P4-5 bp variants |  |  |  |
| (∆27) *pbuE^b^* | 2250±230 | 28900±700 | 13±1 |
| (∆27) P4-5 bp/U | 480±36 | 20900±700 | 45±7 |
| (∆27) P4-A | 270±30 | 26500±700 | 93±7 |
| (∆27) P4-B | 1610±60 | 50600±1800 | 32±1 |
| (∆27) P4-C | 180±20 | 21600±300 | 120±10 |
| (∆27) P4-D | 6970±340 | 15200±800 | 2.3±0.1 |
| (∆27) P4-E | 48700±800 | 51700±1100 | 1.1±0.1 |
| (∆27) P4-F | 29300±1000 | 51000±1000 | 1.8±0.1 |
| (∆27) P4-G | 380±20 | 21600±500 | 59±6 |
|  |  |  |  |

**S2 Table. Data values for Fig 2 – Fig 7 and Fig S1.**

^a^Median vaule of background normalized fluorescence with each measurement represents at least three technical replicates of three independent biological replicates. Errors are reported as standard errors as calculated in Excel.
